# Boosting the epoxidation of squalene to produce triterpenoids in *Saccharomyces cerevisiae*

**DOI:** 10.1186/s13068-023-02310-6

**Published:** 2023-05-04

**Authors:** Meng-Meng Du, Ge-Ge Zhang, Zhan-Tao Zhu, Yun-Qiu Zhao, Bei Gao, Xin-Yi Tao, Feng-Qing Wang, Dong-Zhi Wei

**Affiliations:** State Key Laboratory of Bioreactor Engineering, Newworld Institute of Biotechnology, East China University of Science and Technology, 130 Meilong Road, P.O.B.311, Shanghai, 200237 China

**Keywords:** 2,3-Oxidosqualene, 2,3:22,23-Dioxidosqualene, Triterpenoid, ERG7, Two-stage fermentation

## Abstract

**Background:**

Polycyclic triterpenoids (PTs) are common in plants, and have attracted considerable interest due to their remarkable biological activities. Currently, engineering the ergosterol synthesis pathway in *Saccharomyces cerevisiae* is a safe and cost-competitive way to produce triterpenoids. However, the strict regulation of ERG1 involved in the epoxidation of squalene limits the triterpenoid production.

**Results:**

In this study, we found that the decrease in ERG7 protein level could dramatically boost the epoxidation of squalene by improving the protein stability of ERG1. We next explored the potential factors that affected the degradation process of ERG1 and confirmed that ERG7 was involved in the degradation process of ERG1. Subsequently, expression of four different triterpene cyclases utilizing either 2,3-oxidosqualene or 2,3:22,23-dioxidosqualene as the substrate in ERG7-degraded strains showed that the degradation of ERG7 to prompt the epoxidation of squalene could significantly increase triterpenoid production. To better display the potential of the strategy, we increased the supply of 2,3-oxidosqualene, optimized flux distribution between ergosterol synthesis pathway and β-amyrin synthesis pathway, and modified the GAL-regulation system to separate the growth stage from the production stage. The best-performing strain ultimately produced 4216.6 ± 68.4 mg/L of β-amyrin in a two-stage fed-fermentation (a 47-fold improvement over the initial strain).

**Conclusions:**

This study showed that deregulation of the native restriction in ergosterol pathway was an effective strategy to increase triterpenoid production in yeast, which provided a new insight into triterpenoids biosynthesis.

**Supplementary Information:**

The online version contains supplementary material available at 10.1186/s13068-023-02310-6.

## Background

Polycyclic triterpenoids (PTs) are common in plants, and have attracted considerable interest due to their remarkable biological activities [1, 2]. However, the contents of PTs in plants are generally low and they are often mixed with structural analogues. Thus, the extraction and purification of PTs from plants are usually challenging and unsustainable due to the massive consumption of plant resources and extraction solvents, which inevitably wreaks havoc on the environment [3]. In general, it is not feasible to synthesize them by chemical means because the multiple-fused ring structures and chiral groups of PTs are difficult to be constructed by cost-effective means of stereochemistry [4]. An attractive alternative is to produce them in the engineered microorganisms, which can specifically biosynthesize PTs in a fast and inexpensive way. Among these industrialized microorganisms, the budding yeast *Saccharomyces cerevisiae* has emerged as the engineered platform for PTs synthesis [5–7].

**Fig. 1:**
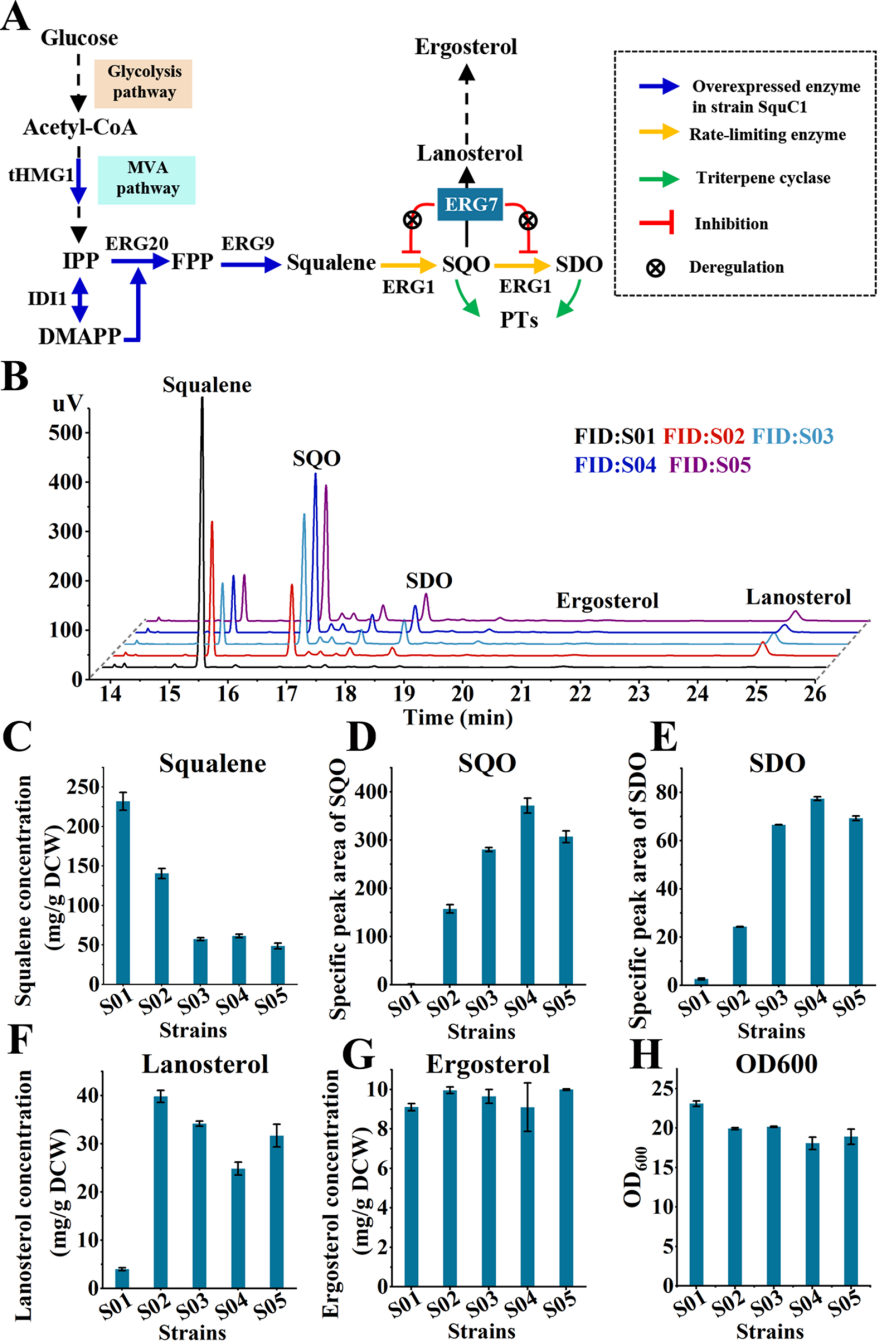
Boosting the epoxidation of squalene by degrading ERG7. **A** Schematic representation of the PT synthesis pathway on the basis of the native ergosterol synthesis pathway in yeast and the revealed negative effect of ERG7 on ERG1. tHMG1, 3-hydroxy-3-methylglutaryl-coenzyme-A reductase 1; IDI1, isopentenyl-diphosphate delta-isomerase; ERG20, FPP synthase; ERG9, squalene synthase; ERG1, squalene monooxygenase; ERG7, lanosterol synthase; IPP, isopentenyl pyrophosphate; DMAPP, Dimethylallyl diphosphate; FPP, farnesyl diphosphate; SQO, 2,3-oxidosqualene; SDO, 2,3:22,23-dioxidosqualene; PTs, Polycyclic triterpenoids. **B** GC chromatograms of ERG7-degraded strains and the control strain S01. **C**–**H** Effect of degrading ERG7 by N-degron-mediated protein degradation strategy on the corresponding metabolites of squalene (**C**), SQO (**D**), SDO (**E**), lanosterol (**F**), ergosterol (**G**) and OD_600_ (**H**). Specific peak area is defined as the peak area in the unit dry cell weight. GC–MS analysis of SQO and SDO are provided in Additional file 1:Fig. S2. All values presented are the means of three biological replicates, and error bars represent standard deviations

It is well known that 2,3-oxidosqualene (SQO) is the direct precursor of most triterpene cyclases (OSCs), which catalyze the first committed step in triterpenoid synthesis pathway (Fig. 1A) [3, 8]. For example, β-amyrin, lupeol, and cucurbitadienol were synthesized from SQO, respectively, under the catalysis of β-amyrin synthase (GgbAS1) from *Glycyrrhiza glabr* [6], lupeol synthase (AtLUS1) from *Arabidopsis thaliana* [9], and cucurbitadienol synthase (SgCS) from *Siraitia grosvenorii* [10]. Recently, 2,3:22,23-dioxidosqualene (SDO) was confirmed as the precursor of several valuable triterpenoids including noncaloric nonsugar sweetener mogroside V [10] and α-onocerin [8]. However, the generation of the two epoxidized squalenes (SO) especially SDO was strictly regulated by two key enzymes (HMGR and ERG1) in *S. cerevisiae*. The two epoxidized squalenes were synthesized through the mevalonate (MVA) pathway, in which the metabolic flux was strictly controlled by the rate-limiting enzyme, 3-hydroxy-3-methylglutaryl-coenzyme-A reductase (HMGR) [11]. Overexpressing a truncated HMGR (tHMG1) that circumvented the regulation by removing its transmembrane domain dramatically increased the metabolic flux of the MVA pathway [12]. Then, two molecules of IPP and one molecule of DMAPP were catalyzed to generate farnesyl diphosphate (FPP) by FPP synthase (ERG20). Two FPP molecules were subsequently coupled with squalene synthase (ERG9) to produce squalene, which was the most easily accumulated intermediate in the engineered triterpenoid synthesis pathway [13], because the enzyme ERG1 catalyzing the conversion of squalene to the two SO was strictly regulated. And the regulatory mechanism on ERG1 has not been fully understood.

The sufficient precursor supply has successfully boosted PT production [7, 13–15]. Therefore, improving the activity of ERG1 is an efficient strategy to boost the production of PTs. In previous studies, the supply of precursor for PT production was usually increased by expressing *Erg1* with a strong promoter and integrating *Erg1* at multi-copy sites [7, 16]. Nonetheless, the ability of over-expressing ERG1 to enhance the conversion of squalene to SQO, especially SDO, is still limited due to its tight regulation. Moreover, the high expression level of *Erg1* not only imposes a burden on microbes, but also significantly increases the content of lanosterol for the subsequent sterol synthesis pathway [17]. In addition, most genes integrated at multi-copy sites cannot be stably inherited [18, 19], thus hindering the industrial application of engineered strains. Therefore, it is urgent to abolish the regulation of ERG1 so as to increase the precursor supply for PT production.

It was reported that ERG1 might be negatively regulated by lanosterol [20]. In this study, the activity of ERG7, responsible for the conversion of SQO to lanosterol, was weakened by the N-degron-mediated protein degradation strategy and thus the epoxidation of squalene catalyzed by ERG1 was significantly boosted. We next analyzed the transcriptional level, protein stability and subcellular location of ERG1, and demonstrated that decreasing the protein level of ERG7 only improved the protein stability of ERG1. Then, we tested potential factors regulating ERG1 including sterols, N-degrons and ERG7, and confirmed that ERG7 might influence the degradation process of ERG1. Subsequently, we applied the strategy in the production of four different triterpenoids and the concentrations (mg/g DCW) of the four different triterpenoids in ERG7-degraded strains were significantly increased compared with the corresponding reference strains. Finally, we further optimized a triterpenoid-producing strain by enhancing the supply of 2,3-oxidosqualene, balancing the flux distribution between ergosterol pathway and β-amyrin pathway, and modifying the GAL-regulation system to separate the growth stage from the production stages. The optimal strain in a two-stage fed-fermentation ultimately produced 4216.6 ± 68.4 mg/L of β-amyrin, which is the highest up to date [21].

## Results and discussion

### Boosting the epoxidation of squalene by degrading ERG7

In *S. cerevisiae*, PT synthesis pathway was engineered based on the native ergosterol synthesis pathway, and ERG1, the checkpoint on the ergosterol synthesis pathway, became the rate-limiting enzyme in the engineered PT synthesis pathway [22, 23]. It was reported that the native promoter of ERG1 was regulated by ergosterol [24]. To remove the regulation, ERG1 placed under the control of the GAL10 promoter (P_GAL10_) was introduced into a squalene-producing strain SquC1 constructed in our previous work by enhancing the upstream flux and modifying the GAL-regulation system through deleting GAL80 and overexpressing GAL4 so as to achieve the massive accumulation of squalene (Fig. 1A, Additional file 1: Table S1) [13]. The content of squalene in strain S01 was slightly lower than that in strain SquC1, indicating that the copy number of ERG1 in strain S01 needed to be further increased (Additional file 1: Fig. S1). However, it was reported that the over-expression of ERG1 could increase the level of lanosterol [17, 23], which would accelerate the degradation of ERG1 to impede the epoxidation of squalene. Therefore, increasing the copy number of ERG1 might accelerate the degradation of ERG1 and made it difficult to increase the activity of ERG1.

To remove the regulation effect of lanosterol on the protein level of ERG1, the concentration of lanosterol synthase (ERG7) should be reduced. ERG7 is a stable protein with a half-life of more than 10 h [25], so it is difficult to be reduced at the transcriptional level. Another method to reduce the concentration of ERG7 is to accelerate the degradation of ERG7. The N-degron-mediated protein degradation strategy is to fuse a degradation signal peptide to the N-terminus of the target protein, which can significantly accelerate the digestion of the protein [26, 27]. This strategy had been employed to reduce the concentration of ERG20 in *S. cerevisiae* [25]. In view of this, four different N-degrons with varying efficiency (K15 < K3K15 < KN119 ≈ KN113) were selected to be tagged at the N-terminus of ERG7 in strain S01 to construct the strains S02, S03, S04 and S05 [25]. As expected, compared with strain S01, strains S02, S03, S04 and S05 showed significant increases in SO contents and especially the SQO content was increased by more than 118 folds, even 279 folds (Fig. 1D, E). Correspondingly, the squalene content in these strains was decreased by more than 40% or even 79% (Fig. 1C). The sharp decrease in squalene content and the obvious increases in SO contents illustrated that the restriction on the epoxidation of squalene catalyzed by ERG1 was obviously alleviated. Intriguingly, the content of lanosterol in ERG7-degraded strains was greatly enhanced by at least fivefold compared with that in strain S01 (Fig. 1F), suggesting that the increased total activity of ERG1 in ERG7-degraded strains was independent of the lanosterol level. The interesting phenomenon exactly supported the scarcity of 2,3-oxidosqualene caused by the strict regulation of ERG1. Therefore, when the supply of 2, 3-oxisqualene for ERG7 was enough, large amounts of lanosterol were accumulated. The substantial accumulation of lanosterol in ERG7-degraded strains also suggested that ERG7 possessed the high specific activity. Therefore, downregulating ERG7 via the N-degron-dependent protein degradation strategy not only improved the epoxidation of squalene catalyzed by ERG1, but also weakened the competition between the native ergosterol biosynthetic pathway and the triterpenoid pathway for SQO.

### Identifying the negative effect of ERG7 on ERG1

**Fig. 2:**
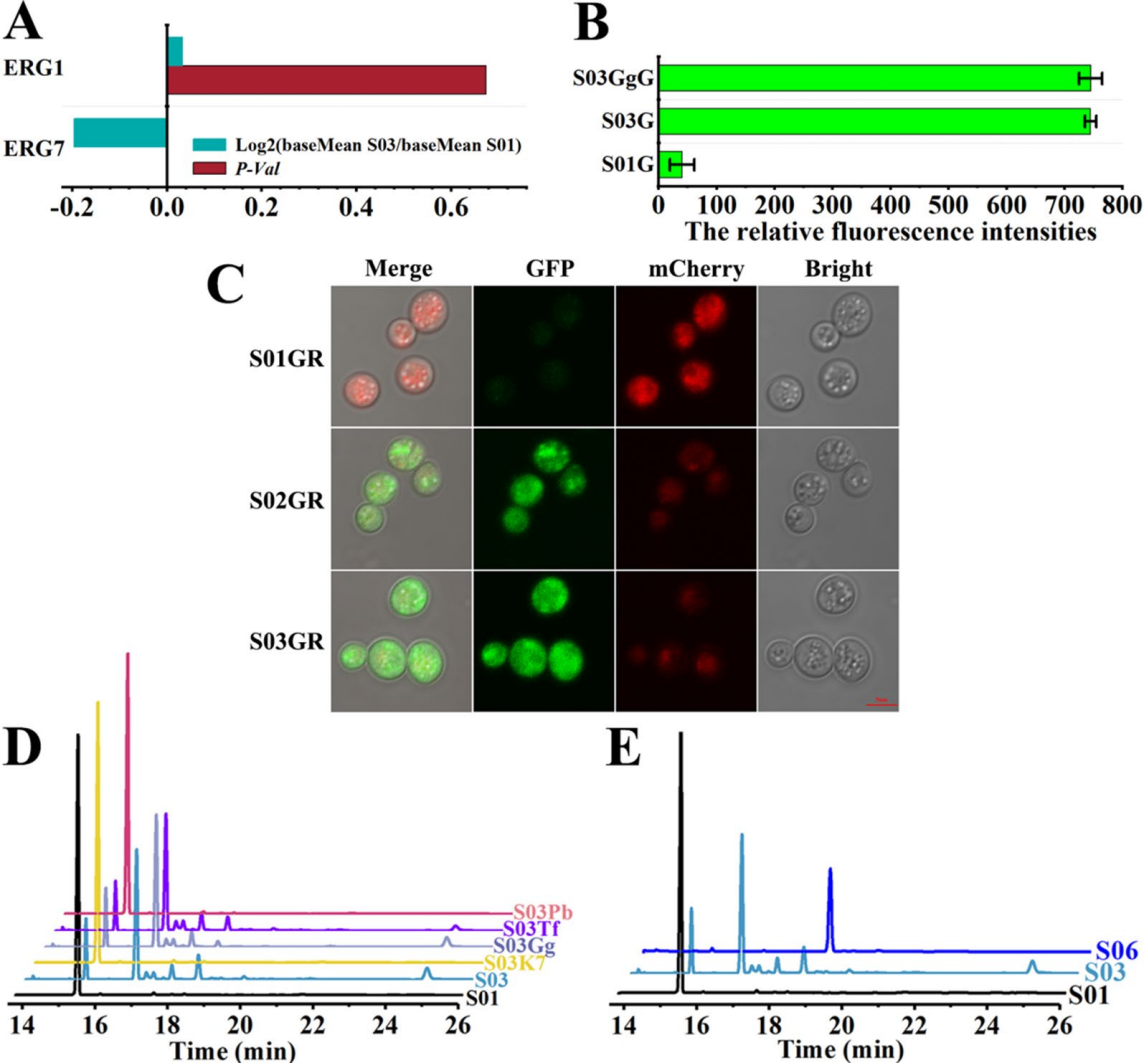
Identifying the regulatory effect of ERG7 on ERG1. **A** The changes in mRNA levels of ERG1 and ERG7 in strain S03 compared to strain S01. The data are obtained by transcriptome sequencing and analyzed using HTSeq (v.0.11.1). When |log2 Fold Change|> 1 and *P*-Val < 0.05, the mRNA level is determined to be significantly upregulated or downregulated. **B** The comparison of ERG1 protein stability in ERG7-degraded strain S03 and the control strain S01 by fluorescence labeling analysis. GFP is fused to the N-terminus of ERG1. **C** The subcellular localization of ERG1 in ERG7-degraded strains and the control strain S01 analyzed by Laser scanning confocal microscopy. GFP is fused to the N-terminus of ERG1 and mCherry is fused to the C-terminus of ERG7. **D** The comparison of GC chromatograms of Erg7-modified strains S03K7, S03Gg, S03Tf, S03Pb and control strains S01 and S03. **E** The GC chromatograms of strains S01, S03 and S06. The concentrations of the corresponding metabolites are provided in Additional file 1: Fig. S3. All values presented are the means of three biological replicates, and error bars represent standard deviations

The above results showed that decreasing ERG7 protein level benefited the epoxidation of squalene, suggesting that the total activity of ERG1 was improved. Previous reports suggested that the transcriptional level, protein stability and subcellular localization of ERG1 affected the activity of ERG1 [20, 24, 28]. The transcriptome sequencing data of a ERG7-degraded strain S03 and the control strain S01 showed that the mRNA level of ERG1 in strain S03 was similar to that in strain S01 (Fig. 2A). The result illustrated that the decrease in the protein level of ERG7 had no effect on the mRNA level of ERG1. The fluorescence labeling analysis showed that the protein level of ERG1 in ERG7-degraded strains was significantly higher than that in the control strain S01, demonstrating that the decrease in the protein level of ERG7 enhanced the protein stability of ERG1 (Fig. 2B). In the laser scanning confocal microscope experiment, we observed that the subcellular localization of ERG1 was not obviously different between the two different ERG7-degraded strains (S02 and S03) harboring the different activities of ERG1 (Fig. 2C). In short, decreasing the protein level of ERG7 affected the degradation process of ERG1 and thus improved the total activity of ERG1 so as to accelerate the epoxidation of squalene.

In the study, we discovered that ERG1 was more stable in ERG7-degraded strains with the massive accumulation of lanosterol compared with the reference strain S01. However, in the earlier study [20], lanosterol was found to accelerate the degradation of ERG1. The contradiction might be ascribed to the positive effect of lanosterol on the expression of ERG7. To investigate the effect, the transcription levels of ERG7 in ERG7-degraded strain S03 and the reference strain S01 were analyzed. The mRNA level of ERG7 in strain S03 was close to that in strain S01, suggesting that mRNA level of ERG7 was not affected by lanosterol. The effect of ERG7 on ERG1 might be independent of lanosterol. Moreover, it was reported that the sterols synthesized in the downstream of lanosterol had no effect on the protein stability of ERG1 [20]. In view of this, N-degron ERG7 might affect the degradation process of ERG1. To investigate whether N-degrons fused to ERG7 impeded the degradation process of ERG1, we over-expressed the N-degron fused ERG7 (K3K15-ERG7) under the control of constitutive promoter TDH3 in strain S03 harboring N-degron K3K15 fused to the N-terminus of the native ERG7. The resulting strain S03K7 accumulated large amounts of squalene and trace amounts of lanosterol and SO (Fig. 2D). In other words, the performance of strain S03K7 was similar to that of strain S01. The result demonstrated that N-degrons fused to ERG7 did not affect the degradation process of ERG1. To investigate whether ERG7 affected the degradation process of ERG1, we selected heterologous lanosterol synthases to replace ERG7. We speculated that if ERG7 affected the degradation process of ERG1, the regulation function was the feature of ERG7 and possibly other lanosterol synthases originated from yeasts. In other words, the lanosterol synthases originated from other species would lose the regulation function on ERG1. In this regard, two lanosterol synthases from *Gallus gallus* (*GgLss1*) and *Talaromyces flavus* (*TfLss1*), and one lanosterol synthases from yeast *Pichia pastoris* (PbLss1) were selected to replace Erg7 of strain S01 in *vivo*, generating strains S03Gg, S03Tf and S03Pb, respectively. As expected, the performances of strains S03Gg and S03Tf in the epoxidation of squalene were similar to that of strain S03, and the performance of strain S03Pb in the epoxidation of squalene was similar to that of strain S01 (Fig. 2D). The result demonstrated that the negative effect of ERG7 on ERG1 was removed in the strains S03Gg and S03Tf, but it was not removed in the strain S03Pb. To further prove that the stability of ERG1 in Erg7-replaced strains was improved, GFP was fused at the N-terminus of ERG1 in strain S03Gg and the fluorescence intensity of the resulting strain S03GgG was detected. The fluorescence intensity of the resulting strain S03GgG was similar to that of strain S03G and significantly higher than that of strain S01G (Fig. 2B), suggesting that the negative effect of ERG7 on ERG1 could be relieved by replacing ERG7 with its heterologous counterpart. The protein sequences of GgLSS1, TfLSS1, and PbLSS1, respectively, had 35%, 42% and 60% identical amino acids with that of ERG7 (Additional file 1: Fig. S4). It is believed that proteins with the higher amino acid sequence identity had the more similar structures [29]. Therefore, the structure of PbLSS1 was more similar to ERG7 among the three ERG7 counterparts, indicating that the regulation effect of ERG7 on ERG1 did not depend on the enzyme activity of ERG7 but the structure of ERG7. The results also suggested that the negative effect of lanosterol synthase on squalene monooxygenase existed not only in *S. cerevisiae* but also in other yeasts.

### Boosting PT production by promoting the epoxidation of squalene

Considering that triterpenoids are usually toxic to cells and that adequate substrate supply is important for the production of triterpenoids, strain S03 was selected as the chassis because of its best growth and higher SQO accumulation among these SQO-producing strains (Fig. 1D, H). However, the content of 2,3:22,23-dioxidosqualene accumulated in strain S03 was too low. In *S. cerevisiae*, 2,3:22,23-dioxidosqualene was converted from 2,3-oxidosqualene by ERG1. To boost the conversion of 2,3-oxidosqualene into 2,3:22,23-dioxidosqualene, we placed the P_GAL10_ controlling the expression of ERG1 with a stronger promoter GAL1 (P_GAL1_) in strain S03. As expected, 2,3-oxidosqualene was successfully converted into 2,3:22,23-dioxidosqualene in the resulting strain S06 (Fig. 2E), illustrating that deregulation of ERG1 at protein level could help unlock the full potential of optimization strategies at the transcriptional level. To be noted, less SDO was accumulated and SQO was substantially accumulated in strain S03. Moreover, the proportion of SDO was significantly increased with the increase in the expression level of ERG1. The phenomenon suggested that ERG1 had the higher affinity to squalene than SQO.

**Fig. 3:**
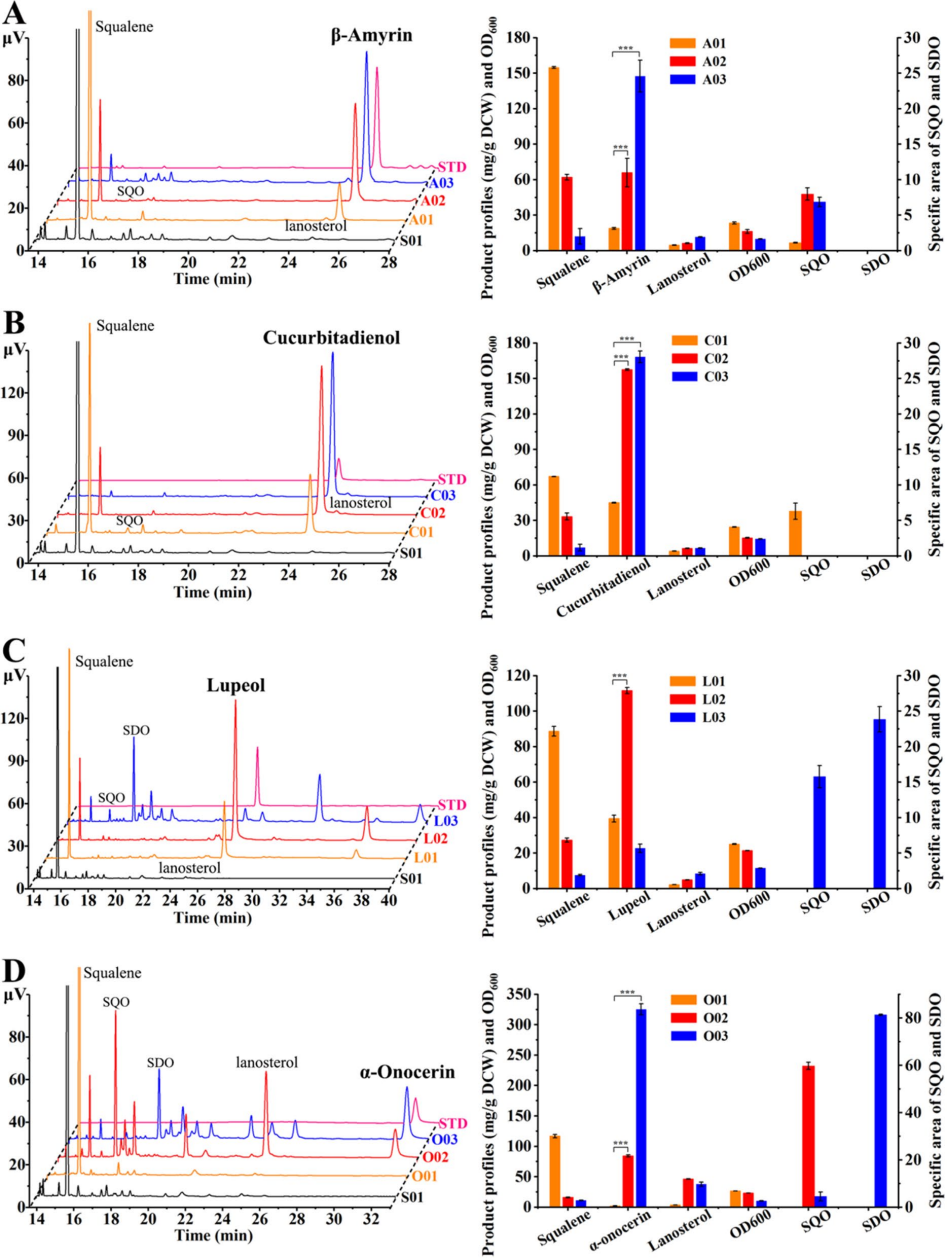
Boosting PTs production by promoting the epoxidation of squalene. The effects of the strategy on the corresponding metabolites of PT, squalene, SQO, SDO, lanosterol and OD_600_ in the β-amyrin-producing strains (**A**), cucurbitadienol-producing strains (**B**), lupeol-producing strains (**C**) and α-onocerin-producing strains (**D**). The titers of each PT are provided in Additional file 1: Fig. S5B. STD is an abbreviation for "Standard". All values presented are the means of three biological replicates, and error bars represent standard deviations. The statistical significance was determined by a Student's *t-test* (***: *p* < 0.001)

To explore whether the deregulation of ERG1 by the strategy could effectively boost the production of PTs, four different plant-derived OSCs including β*-*amyrin synthase (*GgbAs1*) from *Glycyrrhiza glabr* [6], cucurbitadienol synthase (*SgCs*) from *Siraitia grosvenorii* [10], lupeol synthase (*AtLus1*) from *Arabidopsis thaliana* [9] and α-onocerin synthase (*Ons1*) from *Ononis spinosa* [8] were placed under the control of P_GAL1_ and then, respectively, integrated into the reference strain S01 and two epoxidized squalene-producing strains (S03 and S06), thus generating the four groups of PT-producing strains: β-amyrin-producing strains (A01, A02, and A03), cucurbitadienol-producing strains (C01, C02, and C03), lupeol-producing strains (L01, L02, and L03), and α-onocerin-producing strains (O01, O02, and O03) (Additional file 1: Fig. S5A). In the production strains of β-amyrin (Fig. 3A), the concentrations of β-amyrin in strains A02 (65.9 ± 12.0 mg/g DCW) and A03 (147.5 ± 13.5 mg/g DCW) were, respectively, 3.5-fold and 7.8-fold of that in strain A01 (18.8 ± 0.7 mg/g DCW), demonstrating that the strategy could effectively boost the PT production. The concentration of β-amyrin in strain A03 was higher than that in strain A02 due to the enhanced epoxidation of squalene (Fig. 3A). Moreover, SDO was not detected in strain A03 constructed from the strain S06, suggesting that the activity of GgbAS1 might be higher than that of ERG1. GgbAS1 could deplete SQO immediately when squalene was converted into SQO by ERG1, so SDO was difficult to be produced in the strain A03. Therefore, the high expression of ERG1 benefited the epoxidation of squalene and further boosted the β-amyrin synthesis.

In the production strains of cucurbitadienol (Fig. 3B), the concentrations of cucurbitadienol in strains C02 and C03 were similar and higher than that in strain C01. The concentration of cucurbitadienol in strain C03 was the highest and reached 168.4 ± 5.0 mg/g DCW, which was 3.7-fold of that in strain C01 (45.1 ± 0.3 mg/g DCW). The highest titer of cucurbitadienol in strain C03 was 494.0 ± 19.4 mg/L (Fig. S5B), which was the highest production in shake flasks up to date [30]. Similar to β-amyrin, SDO was not detected and only trace amounts of 24, 25-epoxycucurbitadienol, the catalytic product of SDO by SgCS [10], was accumulated in strain C03 (Additional file 1: Fig. S6). The result illustrated that the substrate preferences of ERG1 for squalene allowed triterpene cyclases to catalyze SQO and the high activity of triterpene cyclases depleted SQO as soon as the squalene was depleted by ERG1, thus limiting the generation of SDO. Therefore, the high expression level of ERG1 was conducive to the production of SQO-derived triterpenoids, in which the first committed step of SQO was catalyzed by triterpene cyclases with higher enzymatic activities.

In the production strains of lupeol (Fig. 3C), strain L02 produced the highest concentration of lupeol, 111.7 ± 1.8 mg/g DCW, which was 2.8-fold of that in strain L01 (39.5 ± 1.9 mg/g DCW). The titer of lupeol in strain L02 was the highest and reached 487.5 ± 5.1 mg/L (Additional file 1: Fig. S5B), which was the highest production in shake flasks up to date [31]. However, the lupeol concentration in strain L03 was lower than that in strain L01. Compared with the strains L01 and L02, strain L03 had higher SDO and more by-products accumulation, unlike the production of β-amyrin and cucurbitadienol in the same background strain S06. The accumulation of SDO in strain L03 indicated that SQO was in surplus when squalene was depleted by ERG1, suggesting that the activity of AtLUS1 was lower than ERG1. Therefore, an appropriate expression level of ERG1 to boost the epoxidation of squalene was important for the production of SQO-derived triterpenoids, in which the first committed step of SQO was catalyzed by triterpene cyclases possessing the lower enzymatic activities.

In the production strains of SDO-derived PT α-onocerin (Fig. 3D), strain O03 produced the highest α-onocerin concentration (325.4 ± 9.1 mg/g DCW) (Additional file 1: Fig. S5B), which was 154.9-fold of that in strain O01 (2.1 ± 0.5 mg/g DCW) and 3.9-fold of that in strain O02 (84.4 ± 1.7 mg/g DCW) because the expression of ERG1 placed under the control of a strong promoter contributed to SDO accumulation (Fig. 2E). The titer of α-onocerin in strain O03 was the highest and reached 684.0 ± 19.7 mg/L, which was the highest production in shake flasks up to date. Therefore, the strategy was significant for the construction of yeast cell factories of producing SDO-derived products and the discovery of new enzymes utilizing SDO as a substrate.

In summary, the strategy benefited the production of SQO- and SOD-derived triterpenoids by increasing the precursor supply. In the production of SDO-derived triterpenoids, the high expression level of ERG1 was conducive to the epoxidation of squalene to SDO, thus boosting the production of SDO-derived triterpenoids. In the production of SQO-derived triterpenoids, the strategy showed difference performances because the high expression level of ERG1 not only achieved the complete conversion of squalene, but also competed with triterpene cyclases for SQO. If SQO could be depleted by triterpene cyclases as soon as squalene was depleted by ERG1, the high expression level of ERG1 benefited the production of SQO-derived triterpenoids. If SQO was in surplus when squalene was depleted by ERG1, the protein level of ERG1 should be precisely controlled. In other words, the appropriate protein level of ERG1, which ensured the complete conversion of squalene into SQO and avoided the competition between ERG1 and triterpene cyclases, could significantly improve the production of SQO-derived triterpenoids. In brief, the properties of triterpene cyclases should be taken into consideration when the strategy was applied in the production of SQO-derived triterpenoids.

### Boosting PT production by optimizing the distribution of metabolic flux

**Fig. 4:**
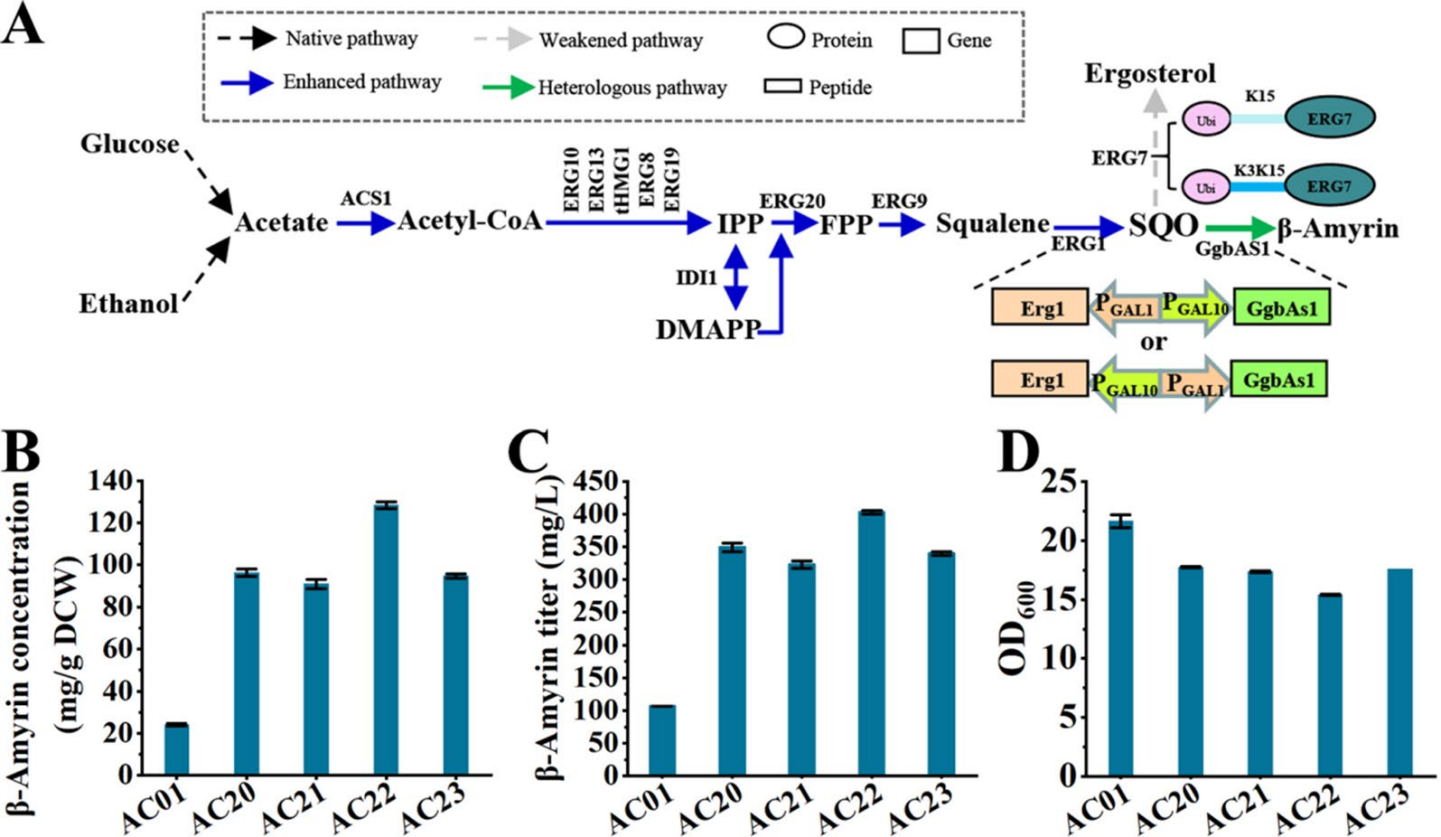
Boosting PT production by optimizing the distribution of metabolic flux. **A** Schematic diagram of pathway optimization in the β-amyrin-producing strain A01. **B**–**D** Effects of enhancing squalene supply and optimizing SQO distribution on β-amyrin concentration (**B**), β-amyrin titer (**C**) and OD_600_ (**D**)

Sterols played an important role in cell growth, so the metabolic flux of the sterol synthesis pathway should be also considered [11]. The best-performing strains in PT production were almost confronted with the insufficient supply of squalene (Fig. 3), indicating that the metabolic flux of the upstream pathway should be enhanced. Here, taking the production of β-amyrin as an example, based on the strain A01, all the genes in the pathway from acetic acid to squalene placed under the control of P_GAL1/10_ were firstly overexpressed to generate strain AC01, which could produce 321.8 ± 20.7 mg/g DCW squalene and 24.1 ± 0.7 mg/g DCW β-amyrin. Given that the conversion of squalene to SQO was influenced by the protein concentration ratio of ERG1 to ERG7 and that the distribution of SQO between sterol synthesis pathway and β-amyrin synthesis pathway was determined by the protein levels of ERG7 and GgbAS1 (Fig. 4A), two N-degrons (K15 and K3K15) for adjusting the protein level of ERG7 together with two combined expression cassettes for adjusting the protein levels of ERG1 and GgbAS1 (T_CYC1_-*Erg1*-P_GAL1_-P_GAL10_-*GgbAs1*-T_ADH1_, T_CYC1_-*GgbAs1*-P_GAL1_-P_GAL10_-*Erg1*-T_ADH1_) were then introduced into strain AC01, thus generating strains AC20, AC21, AC22 and AC23, respectively. Compared with strain A03 (294.0 ± 19.2 mg/L), the best-performing strain AC22 increased the β-amyrin titer to 403.1 ± 2.8 mg/L (128.4 ± 0.5 mg/g DCW), the highest production in shake flasks in current studies (Fig. 4B, C) [21].

### Modifying GAL-regulation system to relieve the conflict between growth and production

**Fig. 5:**
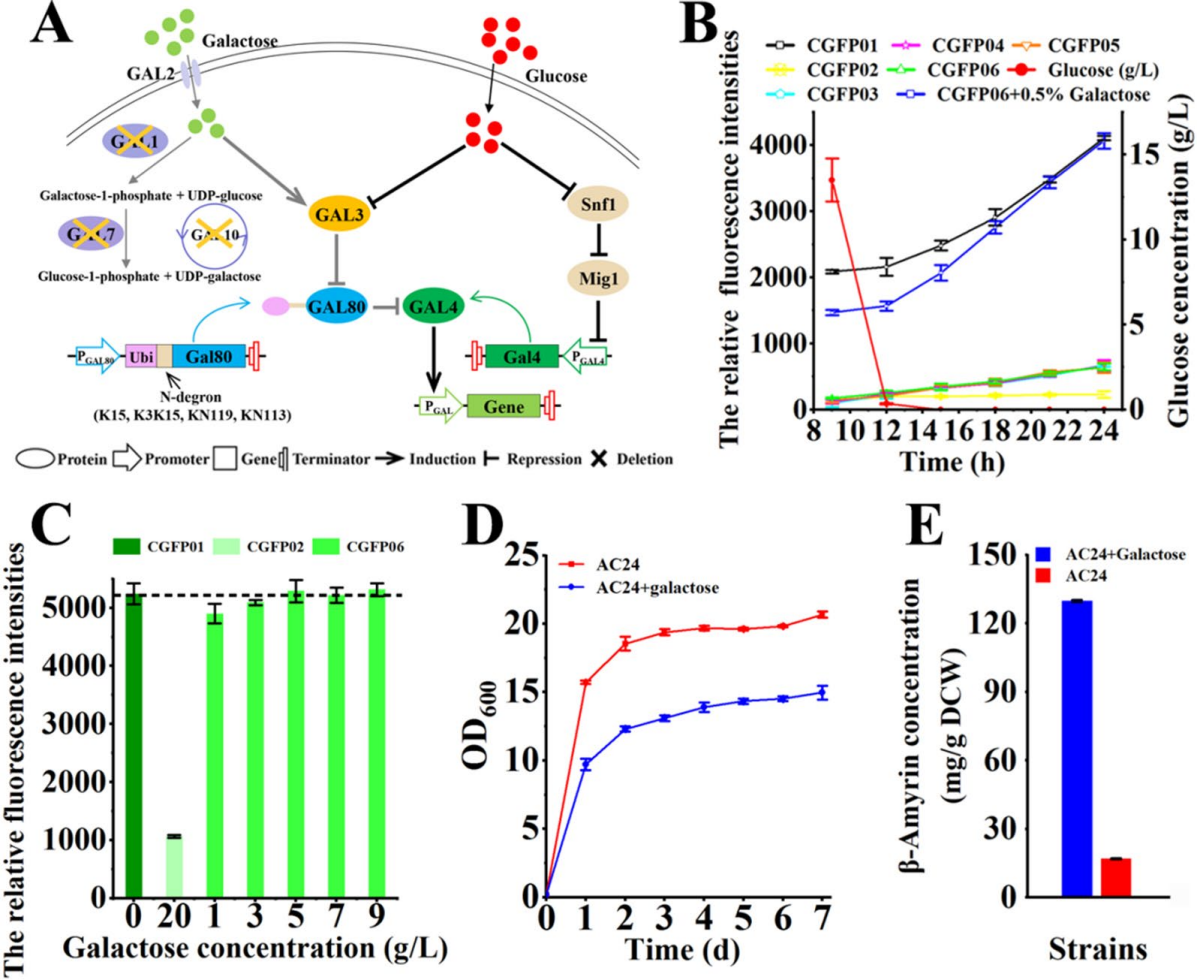
Engineering GAL80 to modify the GAL-regulation system. **A** Schematic diagram of the GAL-regulation system and the Snf1 network in *S. cerevisiae*. **B** Measurements of the leaky expression in different GAL80-engineered strains using GFP as reporter. Strains CGFP01, CGFP02, CGFP03, CGFP04, CGFP05 and CGFP06 were cultured in YPD, and CGFP06 was also cultured in YPD with addition of 5% galactose. **C** Measurements of the transcriptional efficiency in different GAL80-engineered strains using GFP as reporter under conditions of different galactose concentrations. **D** Comparison of the growth state of strain AC24 with or without the addition of 5% galactose. **E** Comparison of β-amyrin production in the strain AC24 with or without the addition of 5% galactose

In *S. cerevisiae*, the native GAL-regulation system is inhibited by the transcriptional repressor GAL80 and activated by GAL4 [32]. The GAL-regulation system in strain SquC1 was modified by knocking out *Gal80*, thus making the transcription of GAL promoters only responsive to glucose concentration. In addition, the galactose metabolic genes *Gal 1/7/10* were also deleted and one copy of *Gal4* under the control of P_*GAL4*_ was introduced to improve the transcriptional efficiency of genes in the engineered pathway (Fig. 5A, Additional file 1: Table S1). However, glucose at low concentrations could not completely block the expression of target genes, thus leading to earlier biosynthesis of the target product [33]. The massive accumulation of β-amyrin in the early stage could cause heavy metabolic burden on cell growth [21], so the GAL-regulation system should be further modified.

GAL80 is the only regulator to repress the transcription of GAL1–GAL10 promoters under low concentrations of glucose and the high protein level of GAL80 needs more galactose to activate the GAL-regulation system, thus increasing the fermentation costs. Due to the long half-life of GAL80 under the galactose induction condition [34], the protein stability of GAL80 was recombined by fusing four N-degrons (K15, K3K15, KN113 and KN119) to its N-terminus to modify the GAL-regulation system. The parent strain CGFP01 was constructed by introducing one copy of *Gfp* under the control of P_GAL1_ into strain SquC1. Then GAL80 and four engineered GAL80 (K15-GAL80, K3K15-GAL80, KN119-GAL80 and KN113-GAL80) were introduced into strain CGFP01, generating strains CGFP02, CGFP03, CGFP04, CGFP05 and CGFP06. The fluorescence intensity in CGFP01 was significantly higher than that in other five strains, demonstrating that the introduction of GAL80 blocked the leaky expression (Fig. 5B). The relative fluorescence intensities in GAL80-engineered strains were similar and slightly higher than that in strain CGFP02 (Fig. 5B), so strain CGFP06 possessing the lowest GAL80 concentration and strain CGFP02 were selected and the transcriptional efficiency of genes in the two strains were compared. When the galactose concentration in the medium reached 5 g/L, the relative fluorescence intensity in strain CGFP06 was close to that in strain CGFP01 (Fig. 5C). However, the relative fluorescence intensity in strain CGFP02 was obviously lower than that in strain CGFP01, although a high galactose concentration (20 g/L) was maintained in the medium (Fig. 5C). Therefore, the engineered GAL80 (KN113-GAL80) might be an optimal option to modify the GAL-regulation system. After the addition of galactose (final concentration = 5 g/L) at the beginning of fermentation, the relative fluorescence intensity in strain CGFP06 was gradually increased close to that in strain CGFP01 after 18 h (Fig. 5B), demonstrating that the addition of galactose could rapidly remove the repression of GAL80, and lead to the timely transcription of GAL1–GAL10 promoters.

To evaluate the effect of the modified GAL-regulation system on PT production, the *KN113-Gal80* expression cassette was introduced into strain AC22, generating strain AC24. Then, the growth state of strain AC24 was measured during shake flask fermentation under galactose or galactose-free conditions. Obviously, the growth state of AC24 under galactose-free conditions was better than that under galactose conditions (Fig. 5D). Moreover, the β-amyrin concentration in strain AC24 under galactose-free condition was significantly lower than that in galactose condition, and the introduction of the engineered GAL80 had no negative effect on PT production (Figs. 5E, 4B), demonstrating that the two-stage fermentation could be achieved by adjusting the addition time of galactose.

### Two-stage controlled high-density fermentation of β-amyrin

**Fig. 6:**
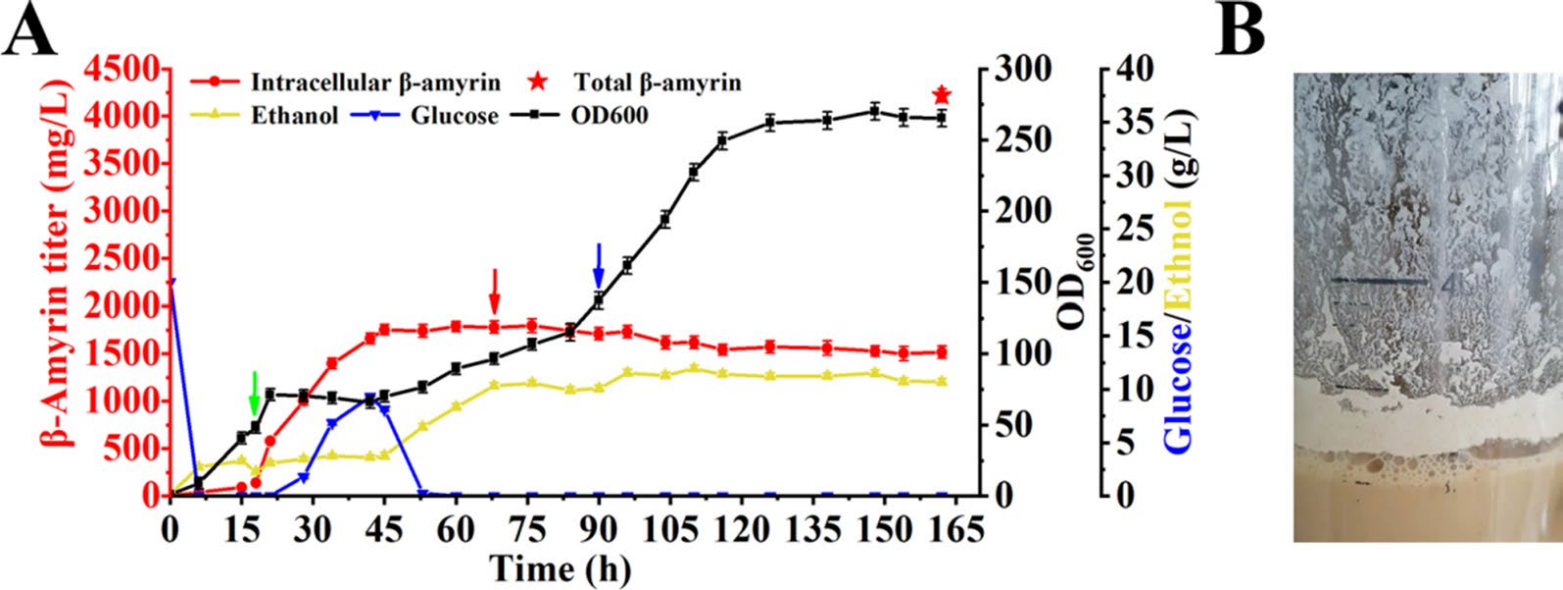
The production of β-amyrin in the two-stage fed-batch fermentation. **A** Fed-batch fermentation of strain AC25. The glucose feeding solution is added at 6^th^ h. Green arrow represents the addition of galactose, red arrow represents the supplementation of absolute ethanol, blue arrow represents the end of glucose supplementation and the absolute ethanol was supplemented until the end of the fermentation. **B** The accumulation of white solids on the tank wall. All values presented are the means of three biological replicates, and error bars represent standard deviations

For the purpose of fed-batch fermentation, strain AC25 was constructed by complementing the auxotrophic markers in AC24. To relieve the significant growth burden caused by the massive accumulation of β-amyrin in the early stage, a two-stage fermentation strategy composed of cell growth stage and β-amyrin accumulation stage was applied (Fig. 6A). Considering that ethanol was conducive to increasing the supply of cytosolic acetyl-CoA for production [35]; we, respectively, added glucose as the main carbon source in cell growth stage and ethanol as the main carbon source in the production stage of β-amyrin. At the beginning of fermentation, the strain grew rapidly and glucose solution was supplemented after 6 h. Due to the strict regulation of GAL-regulation system, β-amyrin was rarely accumulated. After 18 h, the strain entered the logarithmic growth phase and OD_600_ reached about 48. At this time, 15 g of galactose was added to switch on the promoters of GAL1–GAL10. Due to the rapid accumulation of β-amyrin, the strain began to grow slowly (Fig. 6A). After 30-h adaption, the strain began to grow quickly and OD_600_ reached about 96. At this time, absolute ethanol was supplemented as the main carbon source and the feeding rate of glucose solution was gradually reduced. After 90 h, the strain entered the logarithmic growth phase again and absolute ethanol as the sole carbon source was supplemented until the end of the fermentation. In the fermentation process, β-amyrin titer gradually increased within 96 h (up to 1731.6 ± 65.0 mg/L). After that, β-amyrin titer was decreased slightly. The final β-amyrin titer in the fermentation broth was 1516.8 ± 75.0 mg/L at 162 h (Fig. 6A).

Surprisingly, after 70 h, some white solids began to accumulate on the tank wall. The amounts of these white solids increased significantly in the late fermentation stage and β-amyrin titer in the cell remained almost unchanged (Fig. 6). It was thus believed that β-amyrin accumulated in these white solids (Additional file 1: Fig. S7) [36]. After fermentation for 162 h, 3L of ethyl acetate with fermentation broth was added for extraction. The titer of β-amyrin was 4216.6 ± 68.4 mg/L with the concentration of 77.9 ± 13.3 mg/g DCW and the biomass OD_600_ was 265 (Fig. 6A).

## Conclusions

In this work, the regulatory effect of ERG7 on ERG1, the rate-limiting enzyme in triterpenoid pathway, was identified and then a rational metabolic engineering strategy involving deregulation of ERG1 was implemented to improve the production of different triterpenoids derived from SQO or SDO, resulting in 3 to eightfold increase in SQO-derived triterpenoid production and 155-fold increase in SDO-derived triterpenoid production. Subsequently, the β-amyrin-producing strain was selected and the distribution of metabolic flux in the strain was optimized. Finally, the GAL-regulation system was modified to relieve conflict between growth and production. By a two-stage high-density fermentation, the maximum titer of β-amyrin achieved 4216.6 ± 68.4 mg/L with the concentration of 77.9 ± 13.3 mg/g DCW in a 5-L bioreactor. The result represents the highest titer of β-amyrin synthesized in microorganisms.

## Materials and methods

### Strains, media and reagents

The engineered yeasts were kept on YPD medium for culture experiments. Yeast transformants were screened on synthetic complete medium containing an auxotrophic marker (FunGenome, Beijing, China) [37]. 5-Fluoroorotic acid was used for counterselection of yeast transformants. Trans5α Chemically Competent *Escherichia coli* (TransGen Biotech, Beijing, China) was used for plasmid construction and grown in LB medium with 100 μg/mL of ampicillin when required.

### Yeast strains construction

All yeast strains used in this study are listed in Additional file 1: Table S1. The Cas9 expression plasmid pTCL was introduced into strain SquC1 for subsequent gene editing [13]. In the construction of each strain, the amplified upstream homologous arm, the downstream homologous arm, cassettes with at least 50 bp overlapped with the homologous arms, and the gRNA plasmid were co-transformed into the yeast cells with the Frozen-EZ Yeast Transformation II™ Kit (ZYMO RESEARCH, USA) and then plated on SD-Leu-Ura medium. Transformants were directly verified through colony PCR using KOD-FX101 (TOYOBO, Japan). To achieve continuous gene editing, the engineered strains were cross-streaked on an SD-Leu-5-FoA plate to remove gRNA plasmids.

To construct *Erg7* replaced yeast strains, the heterologous lanosterol synthase genes *PbLss1* and *TfLss1* were, respectively, amplified from the genomes of strains *Pichia pastoris* and *Talaromyces flavus* and the heterologous lanosterol synthase gene *GgLss1* was synthesized by GENERY (Shanghai, China) with codon optimization. Then the upstream homologous arm and the downstream homologous arm of *Erg7* were amplified from the genome of CEN.PK2-1C. Finally, the heterologous lanosterol synthase, the homologous arms and the gRNA plasmid pSCM-ERG7C were co-transformed into the yeast cells, and the transformed yeast cells were plated on SD-Leu-Ura medium. The primers used for amplification of the DNA fragments in this study are provided in Additional file 1: Table S3 and sequences of the heterologous lanosterol synthase genes are listed in Additional file 1: Table S4.

### Plasmids construction

All plasmids used in this study are listed in Additional file 1: Table S2. To construct cassettes of all the PTs synthesis pathway genes (*Acs1*, *Erg10*, *Erg13*, *tHmg1*, *Erg12*, *Erg8*, *Erg19*, *Idi1*, *Erg20*, *Erg9*, *Erg1*, Gg*bAs1, AtLus1*, *SgCs* and *Ons1*), traditional restriction enzyme-based cloning was used. gRNA plasmids targeting different genome sites were designed on CRISPRdirect (http://crispr.dbcls.jp/) and constructed using a Gibson assembly cloning kit (Yeason, China). For *Erg10*, *Erg13*, *tHmg1*, *Erg12*, *Erg8*, *Erg19*, *Idi1*, *Erg20*, *Erg9* and *Erg1* genes, the used segments were amplified from the CEN.PK2-1C genome. For Gg*bAs1, AtLus1, SgCs* and *Ons1* genes, the published sequences from *Glycyrrhiza glabra* (Q9MB42.1), *Arabidopsis thaliana* (AT1G78970), *Siraitia grosvenorii* (K7NBZ9.1) and *Ononis spinosa* (KY625496) were acquired from NCBI Database (https://www.ncbi.nlm.nih.gov/) and synthesized by GENERY (Shanghai, China) with codon optimization. The primers used for amplification of the DNA fragments in this study are provided in Additional file 1: Table S3.

### Shake-flask cultivation of engineered yeasts

First, colonies of engineered yeast strains were seeded into 5 mL of YPD liquid medium and cultivated at 30 °C on a rotary shaker (220 rpm) for 18 h. Second, the cultivation solutions were transferred into 15-mL YPD medium at an initial optical density at 600 nm (OD_600 nm_) of 0.1 and then cultivated for approximately 14 h at 30 °C. Finally, the fermentation seeds were inoculated into 50 mL of fresh YPD medium in a 250-mL shake flask at an initial OD_600 nm_ of 0.2 and cultivated at 30 °C for 7d.

### Transcriptomic (RNAseq) analysis

According to the culture method of shake-flask cultivation, strains S01 and S03 were seeded and cultivated. When fermenting for 20 h, strains S01 and S03 were collected by centrifugation for total RNA extraction. Library preparation and sequencing were entrusted to Shanghai Personal Biotechnology Co., Ltd. (Shanghai, China). Three individual samples were sequenced. Raw sequences were quality-filtered and mapped to the CEN.PK2-1C reference genome from Saccharomyces Genome Database (SGD) with the HISAT2 software (http://ccb.jhu.edu/software/hisat2/index.shtml). To examine the correlation of gene expression levels among these two samples, Pearson correlation coefficient was estimated (Additional file 1: Fig. S8). The correlation coefficient between 0.8 and 1 indicated that the similarity of the expression patterns among these two samples was high. In contrast, the correlation was low when correlation coefficient was between 0 and 0.8. Gene expression analysis was performed with HTSeq (v. 0.11.1) and differentially expressed genes (DEGs) were screened according to the conditions of expression difference multiple |log2 Fold Change|> 1 and significant *P*-value < 0.05.

### Fluorescence detection and laser scanning confocal microscopy (LSCM)

Yeast cells containing the GFP gene were cultured in YPD medium at 30 °C for 1d and then collected by centrifugation at 5,000 rpm for 2 min and washed with phosphate buffer (PBS, 100 mM, pH 7.4). To test the relative fluorescence intensity, the samples were diluted with PBS to OD_600_ = 10 and were measured by a SpectraMax M5 microplate reader (excitation 488 nm; emission 520 nm) at sensitivity = 100. To observe the fluorescence changes visually, 10 μL of cell preparations were directly plated on slides and observed at 488 nm and 561 nm with a Nikon A1R confocal laser scanning microscopy (Nikon, Japan).

### Extraction and quantification of triterpenoids

To extract triterpenoids including squalene, 2,3-oxidosqualene, lanosterol, ergosterol, β-amyrin, cucurbitadienol, lupeol and α-onocerin, 600 μL of resuspended cultured cells and 600 μL of ethyl acetate were mixed with 1.5 g of zirconia beads (diameter of 0.5 mm) in 2 mL of microcentrifuge tubes and shaken for 30 min at 55 Hz and − 4 °C with a freeze grinder (Shanghai Jingxin, China). The vibrated mixture was separated by centrifugation at 12,000 rpm for 10 min and the upper organic phase was used for GC detection after dehydration by anhydrous Sodium sulphate. The GC system (Agilent 7820 A, USA) was equipped with an HP-5 capillary column (30 m × 0.25 mm, 0.25 μm film thickness) and a flame ionization detector (FID) using N_2_ (1 mL/min) as the carrier gas. The oven temperature was programmed to rise from an initial temperature of 80 °C for 1 min, gradually increased to 280 °C at a rate of 20 °C/min, maintained for 25 min. Standard 2,3-oxidosqualene, lanosterol, ergosterol, β-amyrin, cucurbitadienol, lupeol and α-onocerin compounds were purchased from BioBioPha (Yunnan, China). To measure the dry cell weight (DCW), an aliquot of the culture (30 mL) was harvested by centrifugation at 5000 × g for 8 min. The collected cells were washed with water and dried to a constant weight by Vacuum freeze dryer (Songyuan, Beijing). We calculated the following relationship between OD_600_ per liter and DCW: One OD_600_ per liter is equal to 0.204 g. All results were reported as the average of three replicates.

### Fed-batch fermentation

To prepare the first seed cultures, a monoclone was picked from YPD plate and inoculated into a 5-mL YPD tube and then cultured at 30 °C for approximately 18 h in a rotary shaker at 220 rpm. The second seed cultures were prepared by transferring 0.15 mL of the first cultures into 250-mL flasks containing 15 mL of YPD and then cultured for approximately 14 h at 220 rpm and 30 °C. The third seed cultures were prepared by transferring 3 mL of the second cultures into 500-mL flasks containing 100 mL of YPD. These cultures were maintained for approximately 24 h in a rotary shaker at 230 rpm and 30 °C, and then 10% (vol/vol) of the seed cultures were inoculated into a 5-L bioreactor (Bai Lun, China) with 2.7 L of medium. The media used for fed-batch fermentation were composed of YPD, 8 g/L KH_2_P0_4_, 3 g/L MgS0_4_, 0.72 g/L ZnS0_4_·7H_2_0, 10 mL/L trace metal solution, and 12 mL/L vitamin solution [38]. Fermentation was carried out at 30 °C and pH was maintained at 5.5 by automatic feeding of 5 M ammonia hydroxide. The dissolved oxygen concentration was maintained above 40% saturation by an agitation cascade (200 − 850 rpm) with an airflow rate of 2 vvm (air volume/working volume/min).

We employed a two-stage fed-batch strategy. At the first stage, a feeding solution containing 500 g/L glucose, 9 g/L KH_2_PO_4_, 2.5 g/L MgSO_4_, 3.5 g/L K_2_SO_4_, 0.28 g/L Na_2_SO_4_, 10 ml/L trace metal solution and 12 ml/L vitamin solution [38], along with 10 g/L yeast extract and 20 g/L peptone as a nitrogen source was used to achieve rapid cell growth. At the second stage, when the strain entered the logarithmic or late-logarithmic growth phase, galactose was added to switch on the β-amyrin biosynthesis pathway. When the cells were adapted and achieved to quickly grow, the feeding solution was changed to absolute ethanol to support β-amyrin accumulation and the ethanol concentration in the fermentation broth was maintained between 5 and 15 g/L. The ethanol concentration constantly monitored by an ethanol electrode (Bai Lun, China).

### Extraction and quantification of total β-amyrin in the Fed-batch fermentation

After fed-batch fermentation, the final volume of fermentation broth was measured as same as the volume of initial fermentation liquid (3L). Next, the fermentation broth was transferred into a 10-L beaker and 3L of ethyl acetate was added. Then, the liquid was stirred by a mixer for 60 min and 1 mL of the mixture was transferred into 2 mL of microcentrifuge tubes with 2.0 g of zirconia beads (diameter of 0.5 mm). According to the method of extraction and quantification of triterpenoids, the total titter of β-amyrin in the fermentation broth was determined.

## Supplementary Information

**Additional file 1: Fig. S1.** Comparison of squalene contents in strains SquC1 and S01. **Fig. S2.** GC–MS analysis of SQO and SDO. **Fig. S3.** The concentrations of each product are provided in ERG7-modified strains. **Fig. S4.** The protein sequences comparison between heterologous lanosterol synthases and ERG7. **Fig. S5.** Removing the bottleneck to stimulate PTs production. **Fig. S6.** Detection of 24,25-epoxycucurbitadienol. **Fig. S7.** Detection of the white solids accumulated on the tank wall during the fed-batch fermentation. **Fig. S8.** Correlation between samples. **Table S1.** *S. cerevisiae* strains used in this work. **Table S2.** Plasmids used in this study. **Table S3.** Primers used in this study. **Table S4.** Heterologous lanosterol synthase sequences.

## Data Availability

All data generated or analyzed during this study are included in this published article and its additional information files.

## Abbreviations

PT: Polycyclic triterpenoid
SO: Epoxidized squalene
SQO: 2,3-Oxidosqualene
SDO: 2,3:22,23-Dioxidosqualene
MVA pathway: Mevalonate pathway
HMGR: 3-Hydroxy-3-methylglutaryl-coenzyme-A reductase
IDI1: Isopentenyl-diphosphate delta-isomerase
ERG20: FPP synthase
ERG9: Squalene synthase
ERG1: Squalene monooxygenase
ERG7: Lanosterol synthase
IPP: Isopentenyl pyrophosphate
DMAPP: Dimethylallyl diphosphate
FPP: Farnesyl diphosphate
LSCM: Laser scanning confocal microscopy
STD: Standard

## References

[1] Augustin JM, Kuzina V, Andersen SB, Bak S (2011). Molecular activities, biosynthesis and evolution of triterpenoid saponins. Phytochem.

[2] Sun HX, Xie Y, Ye YP (2009). Advances in saponin-based adjuvants. Vaccine.

[3] Sawai S, Saito K (2011). Triterpenoid biosynthesis and engineering in plants. Front Plant Sci.

[4] Wu SQ, Chappell J (2008). Metabolic engineering of natural products in plants; tools of the trade and challenges for the future. Curr Opin Biotech.

[5] Jin CC, Zhang JL, Song H, Cao YX (2019). Boosting the biosynthesis of betulinic acid and related triterpenoids in *Yarrowia lipolytica* via multimodular metabolic engineering. Microb Cell Fact.

[6] Zhu M, Wang C, Sun W, Zhou A, Wang Y, Zhang G, Zhou X, Huo Y, Li C (2018). Boosting 11-oxo-beta-amyrin and glycyrrhetinic acid synthesis in *Saccharomyces cerevisiae* via pairing novel oxidation and reduction system from legume plants. Metab Eng.

[7] Wang P, Wei W, Ye W, Li X, Zhao W, Yang C, Li C, Yan X, Zhou Z (2019). Synthesizing ginsenoside Rh2 in *Saccharomyces cerevisiae* cell factory at high-efficiency. Cell Discov..

[8] Almeida A, Dong L, Khakimov B, Bassard J-E, Moses T, Lota F, Goossens A, Appendino G, Bak S (2018). A single oxidosqualene cyclase produces the seco-triterpenoid α-onocerin. Plant physiol.

[9] Segura MJR, Meyer MM, Matsuda SPT (2000). Arabidopsis thaliana LUP1 converts oxidosqualene to multiple triterpene alcohols and a triterpene diol. Org Lett.

[10] Itkin M, Davidovich-Rikanati R, Cohen S, Portnoy V, Doron-Faigenboim A, Oren E, Freilich S, Tzuri G, Baranes N, Shen S (2018). The biosynthetic pathway of the nonsugar, high-intensity sweetener mogroside V from *Siraitia grosvenorii*. P Natl Acad Sci USA.

[11] Hu Z, He B, Ma L, Sun Y, Niu Y, Zeng B (2017). Recent Advances in Ergosterol Biosynthesis and Regulation Mechanisms in *Saccharomyces cerevisiae*. Indian J Microbiol.

[12] Gardner R, Cronin S, Leader B, Rine J, Hampton R, Leder B (1998). Sequence determinants for regulated degradation of yeast 3-hydroxy-3-methylglutaryl-CoA reductase, an integral endoplasmic reticulum membrane protein. Mol biol cell.

[13] Zhu ZT, Du MM, Gao B, Tao XY, Zhao M, Ren YH, Wang FQ, Wei DZ (2021). Metabolic compartmentalization in yeast mitochondria: Burden and solution for squalene overproduction. Metab Eng.

[14] Huang JJ, Zha WL, An TY, Dong H, Huang Y, Wang D, Yu RM, Duan LX, Zhang XL, Peters RJ (2019). Identification of RoCYP01 (CYP716A155) enables construction of engineered yeast for high-yield production of betulinic acid. Appl Microbiol Biot.

[15] Zhao YJ, Fan JJ, Wang C, Feng XD, Li C (2018). Enhancing oleanolic acid production in engineered *Saccharomyces cerevisiae*. Bioresour Technol.

[16] Qu LS, Xiu X, Sun GY, Zhang CY, Yang HQ, Liu YF, Li JH, Du GC, Lv XQ, Liu L (2022). Engineered yeast for efficient de novo synthesis of 7-dehydrocholesterol. Biotechnol Bioeng.

[17] Veen M, Stahl U, Lang C (2003). Combined overexpression of genes of the ergosterol biosynthetic pathway leads to accumulation of sterols in *Saccharomyces cerevisiae*. FEMS Yeast Res.

[18] Wang X, Wang Z, Da Silva NA (1996). G418 selection and stability of cloned genes integrated at chromosomal δ sequences of *Saccharomyces cerevisiae*. Biotechnol Bioeng.

[19] Parekh RN, Shaw MR, Wittrup KD (1996). An integrating vector for tunable, high copy, stable integration into the dispersed Ty δ sites of *Saccharomyces cerevisiae*. Biotechnol prog.

[20] Foresti O, Ruggiano A, Hannibal-Bach HK, Ejsing CS, Carvalho P (2013). Sterol homeostasis requires regulated degradation of squalene monooxygenase by the ubiquitin ligase Doa10/Teb4. Elife.

[21] Du M-M, Zhu Z-T, Zhang G-G, Zhao Y-Q, Gao B, Tao X-Y, Liu M, Ren Y-H, Wang F-Q, Wei D-Z (2021). Engineering *Saccharomyces cerevisiae* for hyperproduction of β-amyrin by mitigating the inhibition effect of squalene on β-amyrin synthase. J Agr Food Chem.

[22] Zhao FL, Bai P, Nan WH, Li DS, Zhang CB, Lu CZ, Qi HS, Lu WY (2019). A modular engineering strategy for high-level production of protopanaxadiol from ethanol by *Saccharomyces cerevisiae*. AIChE J.

[23] Zhang G, Cao Q, Liu J, Liu B, Li J, Li C (2015). Refactoring β-amyrin synthesis in *Saccharomyces cerevisiae*. AIChE J.

[24] Leber R, Zenz R, Schrottner K, Fuchsbichler S, Puhringer B, Turnowsky F (2001). A novel sequence element is involved in the transcriptional regulation of expression of the ERG1 (squalene epoxidase) gene in *Saccharomyces cerevisiae*. Eur J Biochem.

[25] Peng BY, Nielsen LK, Kampranis SC, Vickers CE (2018). Engineered protein degradation of farnesyl pyrophosphate synthase is an effective regulatory mechanism to increase monoterpene production in *Saccharomyces cerevisiae*. Metab Eng.

[26] Varshavsky A (1996). The N-end rule: functions, mysteries, uses. P Natl Acad Sci.

[27] Varshavsky A (2019). N-degron and C-degron pathways of protein degradation. P Natl Acad Sci.

[28] Leber R, Landl K, Zinser E, Ahorn H, Spok A, Kohlwein SD, Turnowsky F, Daum G (1998). Dual localization of squalene epoxidase, Erg1p, in yeast reflects a relationship between the endoplasmic reticulum and lipid particles. Mol Biol Cell.

[29] Giorgetti A, Raimondo D, Miele AE, Tramontano A (2005). Evaluating the usefulness of protein structure models for molecular replacement. Bioinformatics.

[30] Li S-L, Wang D, Liu Y, Lin T-T, Tang J-L, Hua E-B, Zhang X-L, Dai Z-B, Huang L-Q (2017). Study of heterologous efficient synthesis of cucurbitadienol. China J Chin Materia Med.

[31] Zhang J-L, Bai Q-Y, Peng Y-Z, Fan J, Jin C-C, Cao Y-X, Yuan Y-J (2020). High production of triterpenoids in *Yarrowia lipolytica* through manipulation of lipid components. Biotechnol biofuels.

[32] Johnston M, Flick JS, Pexton T (1994). Multiple mechanisms provide rapid and stringent glucose repression of GAL gene expression in *Saccharomyces cerevisiae*. Mol Cell Biol.

[33] Zhou P, Xie W, Yao Z, Zhu Y, Ye L, Yu H (2018). Development of a temperature-responsive yeast cell factory using engineered Gal4 as a protein switch. Biotechnol Bioeng.

[34] Ang K, Ee G, Ang E, Koh E, Siew WL, Chan YM, Nur S, Tan YS, Lehming N (2012). Mediator acts upstream of the transcriptional activator Gal4. PLoS biol.

[35] Westfall PJ, Pitera DJ, Lenihan JR, Eng D, Paddon CJ (2012). Production of amorphadiene in yeast, and its conversion to dihydroartemisinic acid, precursor to the antimalarial agent artemisinin. P Natl Acad Sci USA.

[36] Ahmed MS, Sehgal SA, Tahir RA, Li C (2019). Identification of effective membrane efflux transporters against β-amyrin through molecular docking approach. Chem Technol Biotechnol.

[37] Reider Apel A, d'Espaux L, Wehrs M, Sachs D, Li RA, Tong GJ, Garber M, Nnadi O, Zhuang W, Hillson NJ (2017). A Cas9-based toolkit to program gene expression in *Saccharomyces cerevisiae*. Nucleic Acids Res.

[38] van Hoek P, de Hulster E, van Dijken JP, Pronk JT (2000). Fermentative capacity in high-cell-density fed-batch cultures of baker's yeast. Biotechnol Bioeng.

