# Supplementary material for: Boosting the epoxidation of squalene to produce triterpenoids in Saccharomyces cerevisiae
Source: Biotechnol Biofuels Bioprod. 2023 May 4;16:76. doi: 10.1186/s13068-023-02310-6 (PMC10161426; doi:10.1186/s13068-023-02310-6)
Supplement: Supplementary file 1 — Additional file 1: Fig. S1. Comparison of squalene contents in strains SquC1 and S01. Fig. S2. GC–MS analysis of SQO and SDO. Fig. S3. The concentrations of each product are provided in ERG7-modified strains. Fig. S4. The protein sequences comparison between heterologous lanosterol synthases and ERG7. Fig. S5. Removing the bottleneck to stimulate PTs production. Fig. S6. Detection of 24,25-epoxycucurbitadienol. Fig. S7. Detection of the white solids accumulated on the tank wall during the fed-batch fermentation. Fig. S8. Correlation between samples. Table S1. S. cerevisiae strains used in this work. Table S2. Plasmids used in this study. Table S3. Primers used in this study. Table S4. Heterologous lanosterol synthase sequences. [file 13068_2023_2310_MOESM1_ESM.docx]

**Supplementary material for**

**Boosting the epoxidation of squalene to produce triterpenoids in *Saccharomyces cerevisiae***

Meng-Meng Du, Ge-Ge Zhang, Zhan-Tao Zhu, Yun-Qiu Zhao, Bei Gao, Xin-Yi Tao, Feng-Qing Wang* and Dong-Zhi Wei*

State Key Laboratory of Bioreactor Engineering, Newworld Institute of Biotechnology, East China University of Science and Technology, 130 Meilong Road, Shanghai 200237, China

*Corresponding authors. Address: East China University of Science and Technology, P.O.B.311, 130 Meilong Road, Shanghai 200237, China. Fax: +8621 64250068

E-mail address:

fqwang@ecust.edu.cn (F. Wang)

dzhwei@ecust.edu.cn (D. Wei)

**Contents:**

- Fig. S1-S8
- Table S1-S4

# Supplementary Figures


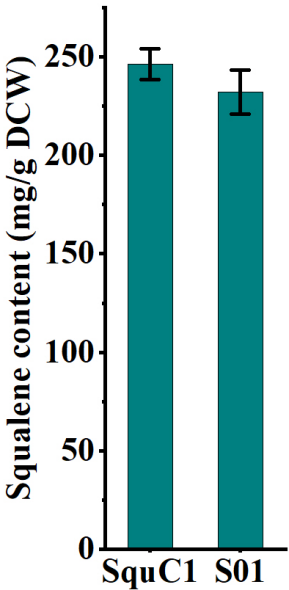


# Fig. S1 Comparison of squalene contents in strains SquC1 and S01.


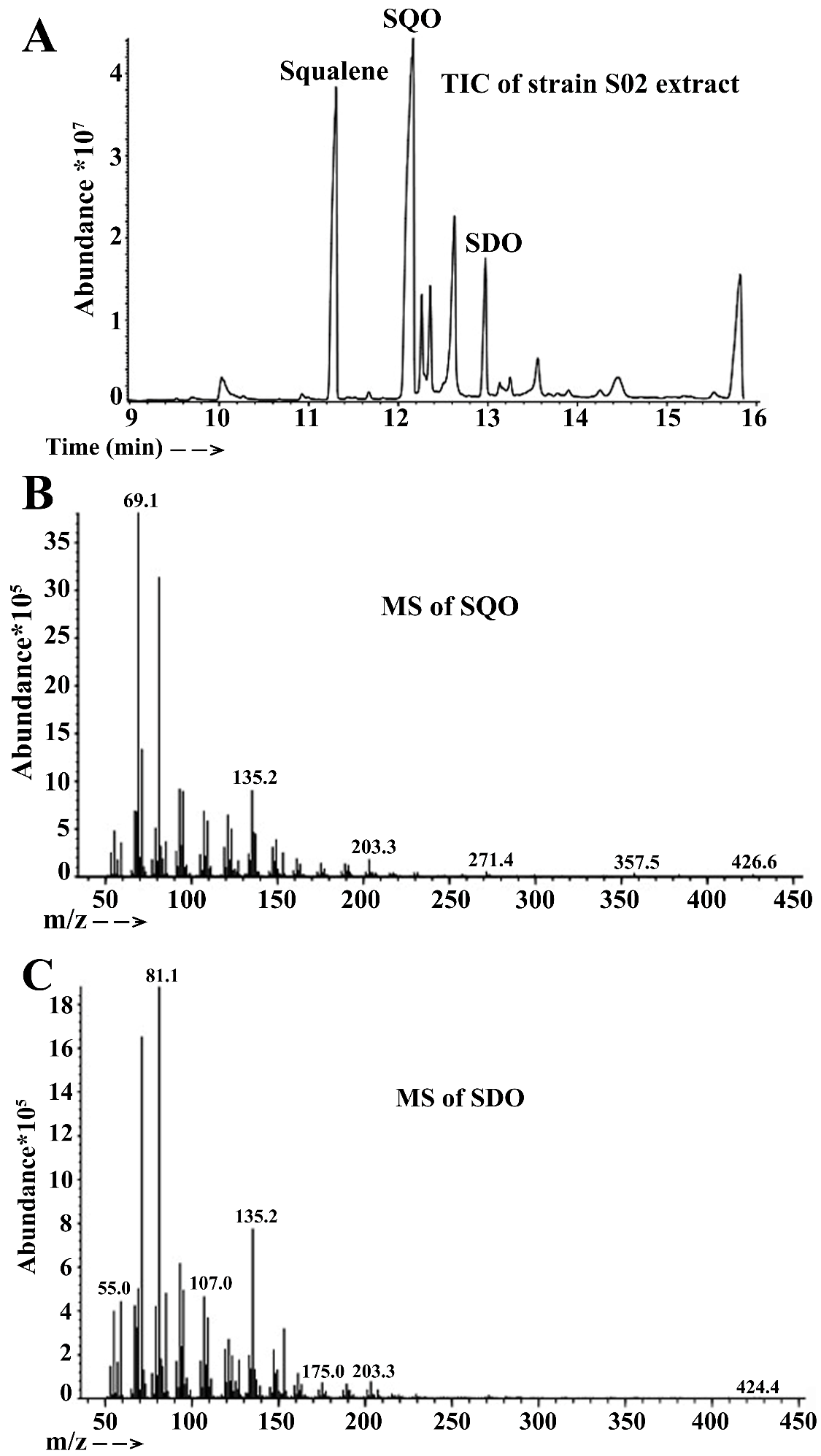


# Fig. S2 GC-MS analysis of SQO and SDO. (A) Total ion chromatogram. Mass spectrum of SQO (B) and SDO (C).


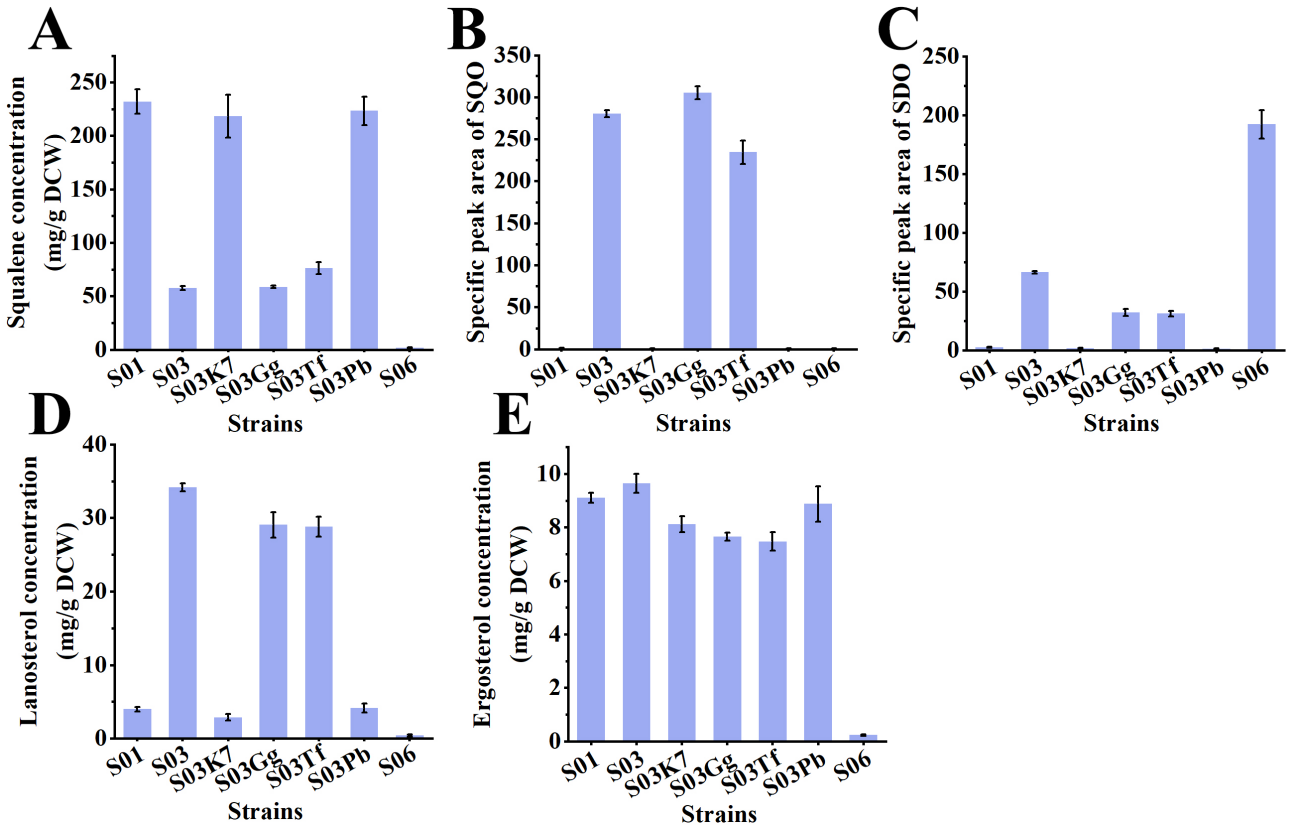


# Fig. S3 The concentrations of each product are provided in ERG7-modified strains. (A) Squalene concentration, (B) Specific peak area of SQO, (C) Specific peak area of SDO, (D) lanosterol concentration and (E) ergosterol concentration.

Upper line: ERG7, from 33 to 721 Lower line: GgLSS1, from 38 to 725

identity= 35.64% (258/724) gap=2.43% (18/742)

1 .....MTEFYSDTIGLPKTDPRLWRLRTDELGRESWEYLTPQQAANDPPSTFTQWLLQDP

:. ::. : ..: : | ||:.| ||... ..:::::. . ::

1 MAADGVVRRRGGPWRTAAATALPSWRLRCEGGRQRWCYLDDGVEDEEAAGGRRAQTALEA

56 KFPQPHPERNKHSPDFSAFDACHNGASFFKLLQEPDSGIFPCQYKGPMFMTIGYVAVNYI

. .. ..: :. . .: . . .:. . ...: .. |. :. :. : : .

61 HSVGLDTDALLRTLPAAGTAREAARNGMRFYATLQAEDGHWAGDYGGPLFLLPGLLIVCH

116 AGIEIPEHERIELIRYIVNTAHPVDGGWGLHSVDKSTVFGTVLNYVILRLLGLPKDHPVC

.: ..: : .:...: .||||||| ||||||||.|||| ||:|||..|.|

121 TARIPLPDGFRREMVRYLRSVQLPDGGWGLHVEDKSTVFGTALNYVALRILGLGPDDPDI

176 AKARSTLLRLGGAIGSPHWGKIWLSALNLYKWEGVNPAPPETWLLPYSLPMHPGRWWVHT

.:|| .| . |||:| | |||:||..||:|.|||:|. || |||| :| ||:|:|.|.

181 VRARVNLHSKGGAVGIPSWGKFWLAVLNVYSWEGMNTLLPEMWLLPTWFPAHPSRLWCHC

236 RGVYIPVSYLSLVKFSCPMTPLLEELRNEIYTKPFDKINFSKNRNTVCGVDLYYPHSTTL

|.||:|:|| ::|.. ..|: .|..|:|...:..|::. .||.|.:.|:| ||| |

241 RQVYLPMSYCYAKRLSAEEDELIRSLQQELYVQDYASIDWPAQRNNVAACDVYTPHSWLL

296 NIANSLVVFYEKYLRNRFIYSLSKKKVYDLIKTELQNTDSLCIAPVNQAFCALVTLIEEG

.|| .:: .|| .. : . .. || ||.: . |..:.|:|:...: || :: :|

301 GIAYAIMNVYEAHHSTYLRQRAITEL.YDHIKADDRFTKCISIGPISKTINMLVRWFVDG

356 VDSEAFQRLQYRFKDALFHGPQGMTIMGTNGVQTWDCAFAIQYFFVAGLAERPEFYNTIV

:|.||| |:.| |: | :||.: |||| | ||.|||:| |: |: .. ||| ..:

360 ENSPAFQEHVSRIPDYLWLGLDGMKMQGTNGSQLWDTAFAVQAFLEAEAQKIPEFMSCLQ

416 SAYKFLCHAQFDTECVPGS..YRDKRKGAWGFSTKTQGYTVADCTAEAIKAIIMVKNSPV

.| .|| .|:..:... ||...||::.|||:. |:.|||||||::|.|:::.:...

420 NAHEFLRFTQIPENPPDYQKYYRHMNKGGFPFSTRDCGWIVADCTAEGLKSIMLLQEKCP

474 FSEVHHMISSERLFEGIDVLLNLQNIGSFEYGSFATYEKIKAPLAMETLNPAEVFGNIMV

| . .. ||||::::|||.:.| :: |||||. ::. :| |||.||||:||:

480 FIANPVPA..ERLFDAVNVLLSMKNSDGG....FATYETKRGGHLLELLNPSEVFGDIMI

534 EYPYVECTDSSVLGLTYFHKYFDYRKE.EIRTRIRIAIEFIKKSQLPDGSWYGSWGICFT

:|.|||||.. : :| |:. :. ::. |||. :. :::| :|.| :|||| ||||:|||

534 DYTYVECTSAVMQALRHFQDVYPEHRAPEIRETLQKGLDFCRKKQQADGSWEGSWGVCFT

593 YAGMFALEALHTVGETYENSSTVRKGCD...FLVSKQMKDGGWGESMKSSELHSYVDSEK

|:. |:|||: .:...| :: ..|... ||:||||.||||||...|.| :.||:|..

594 YGTWFGLEAFASMQHVYRDGVACREVARACQFLLSKQMTDGGWGEDFESCEQRTYVQSST

650 SLVVQTAWALIALLFAEYPNKEVIDRGIDLLKNRQEESGEWKFESVEGVFNHSCAIEYPS

| :..|.|||::|: . ||:.:|::|||.|| ::| ..|:|. |.|.||||.||||.|..

654 SQIHNTCWALLGLMAVRYPDTGVLERGIKLLIDKQLPNGDWPQENVAGVFNKSCAISYTA

710 YRFLFPIKALGMYSRAYETHTL

|| :||| .||.:|| .. .|

714 YRNVFPIWTLGRFSRLHPNSPLAEHLQSRPLAGVGKTPKTALSD

Upper line: ERG7, from 13 to 721 Lower line: TfLSS1, from 30 to 728

identity= 42.68% (306/717) gap=4.65% (35/752)

1 .................MTEFYSDTIGLPKTDPRLWRLRTDELGRESWEYLTPQQAANDP

..: .|. | .||| |||:.:: || . ..|.

1 MAKESKIRPIANWRTNANGHLTKDAAGDDKTDYARWRLHDNDGR......LTWRYLESDE

44 PSTFTQWLLQDPKFPQPHPERNKHSPDFSAFDACHNGASFFKLLQEPDSGIFPCQYKGPM

... . : |. . ..: . ... .::||. || .||. || |.: . |||

55 ENEKWPQTFYDKYNLGLPTGAPELPKAKTPLDAAINGLEFFSKLQMPTGHWACEYG.GPM

104 FMTIGYVAVNYIAGIEIPEHERIELIRYIVNTAHPVDGGWGLHSVDKSTVFGTVLNYVIL

|: | | . ||....||.. :|: ||:.. .:||||||||| :.|..||||:.||||

114 FLLPGVVITWYITNTPIPPEYAVEIKRYLFARQNPVDGGWGLHIEGHSSAFGTVMTYVIL

164 RLLGLPKDHPVCAKARSTLLRLGGAIGSPHWGKIWLSALNLYKWEGVNPAPPETWLLPYS

|||| ..:.| |||: :||||: .|||:|:||| |.: .|...||.||| ||||

174 RLLGASEEDPRMIKARGFAHKLGGALYAPHWAKVWLSLLGVMDWSCANPVPPELWLLPDW

224 LPMHPGRWWVHTRGVYIPVSYLSLVKFSCPMTPLLEELRNEIYTKPFDKINFSKNRNTVC

:|: | |||:| | |::|:||| |: | ..| :||||||..|::.|:|..:||.:.

234 VPIAPYRWWIHMRMVFLPMSYLWSKKWVFPQNELTRQLRNEIYAQPYESIDFASHRNSIA

284 GVDLYYPHSTTLNIANS.LVVFYEKYLRNRFIYSLSKKKVYDLIKTELQNTDSLCIAPVN

| |||.. ||:.|. || .:...|| . : . ... |::||: | :||| .:|||.

294 KEDNYYPKTMFLNVVNTLLVNVWTPLLRFSALAKKAEDWVWELIRMEDENTDYAGLAPVS

343 QAFCALVTLIEEGVDSEAFQRLQYRFKDALFHGPQGMTIMGTNGVQTWDCAFAIQYFFVA

.:: :...|.:| :||...: . :.: |: .:|| ||||.|.||.|| .| . ||

354 NPLNFVCCYIHDGEGSESVRKHREALHEYLWMKGEGMLCNGTNGAQVWDTAFITQAVSVA

403 GLAERPEFYNTIVSAYKFLCHAQFDTECVPGS..YRDKRKGAWGFSTKTQGYTVADCTAE

|:|| |.: :..|..|| : |: .:.. .. ||:.|||||.||.|.|||||.|||||

414 GFAEDPKWRPMLTKALEFLDNHQLRENVPNQDKCYRQHRKGAWPFSNKVQGYTVSDCTAE

461 AIKAIIMVKNSPVFSEVHHMISSERLFEGIDVLLNLQNIGSFEYGSFATYEKIKAPLAME

:::.:: :.: ..|..:. || :::|.:| ||| .: : ||. ::. :|

474 GLRSVLQLQEIHGFPKLVSAD...RLKDAVDCILLLQNSTGGFSE....YESRRGSPLLE

521 TLNPAEVFGNIMVEYPYVECTDSSVLGLTYFHKYF.DYRKEEIRTRIRIAIEFIKKSQLP

||:|||||.||:.|. ||||..|: ::..| ::: ||| |||:. : |:|:||: | |

527 WLNAAEVFGGIMISYDHVECTTASITAMSLFSRFYPDYRAEEIKAAKHKAVEYIKRVQKP

580 DGSWYGSWGICFTYAGMFALEALHTVGETYENSSTVRKGCDFLVSKQMKDGGWGESMKSS

||||||.||||:|||::||||.| .|||||..|.. |:||:||:|||..||||||| ||

587 DGSWYGNWGICYTYAALFALESLSSVGETYSTSDSSRRGCEFLLSKQKEDGGWGESYLSS

640 ELHSYVDSEKSLVVQTAWALIALLFAEYPNKEVIDRGIDLLKNRQEESGEWKFESVEGVF

||| |.: |.| ||||||. :.|: |:||:.|.| |||.||..||:..||| ||:||||

647 ELHVYTQHEMSQVVQTAWVCLSLMEADYPDPEPIRRGIKLLMSRQQANGEWLQESIEGVF

700 NHSCAIEYPSYRFLFPIKALGMYSRAYETHTL

| || |.||.|:|.:||:|||::|. |:...|

707 NMSCMISYPNYKFYWPIRALGLFSQKYGNEALF

Upper line: ERG7, from 5 to 724 Lower line: PbLSS1, from 5 to 697

identity= 60.63% (425/701) gap=3.71% (27/728)

1 MTEFYSDTIGLPKTDPRLWRLRTDELGRESWEYLTPQQAANDPPSTFTQWLLQDPKFPQP

|| :||:.||||||||. ||||.:||||: |:|:..:: |||...:.|:||.:..|. |

1 MTVYYSEKIGLPKTDPQRWRLRVNELGRQYWDYIEKEDLKNDPQTPYVQYLLKGDEFECP

61 HPERNKHSPDFSAFDACHNGASFFKLLQEPDSGIFPCQYKGPMFMTIGYVAVNYIAGIEI

||: . . : . | : . ||:|||||||||||.||||.. |:....|

61 IPEKPQSAFESARNCADFLALIQDE......SGVFPCQYKGPMFMSIGYVVACYFTNTPI

121 PEHERIELIRYIVNTAHPVDGGWGLHSVDKSTVFGTVLNYVILRLLGLPKDHPVCAKARS

|:| |.|:|||:||||||||||||||. ||||.|||.:|||:|||||||||:||| |||.

115 PDHVRTEMIRYVVNTAHPVDGGWGLHEWDKSTCFGTCMNYVVLRLLGLPKDNPVCIKARK

181 TLLRLGGAIGSPHWGKIWLSALNLYKWEGVNPAPPETWLLPYSLPMHPGRWWVHTRGVYI

.| ||||::.| ||| ||| ||:|||||||||||| | |||||.:||.|||||||:: :

175 VLHALGGALATPYWGKAWLSLLNVYKWEGVNPAPPEMWNLPYSLKIHPCRWWVHTRAIAL

241 PVSYLSLVKFSCPMTPLLEELRNEIYTKPFDKINFSKNRNTVCGVDLYYPHSTTLNIANS

|:||:| | |:||||.||||||: ..||.|:|||:||.|||:||||||.. |::|||

235 PLSYISSYKSQMPLTPLLKELRNEIFLQDFDTIDFSKHRNNVCGIDLYYPHTSLLDFANS

301 LVVFYEKYLRNRFIYSLSKKKVYDLIKTELQNTDSLCIAPVNQAFCALVTLIEEGVDSEA

::| |:|. ..::: . .. ||:|||.||.||: |||||||.|| .:|..:|||.:| .

295 ILVGYDKIRPTWLLKESNDA.VYELIKKELANTEHLCIAPVNAAFNTIVAYLEEGPESYN

361 FQRLQYRFKDALFHGPQGMTIMGTNGVQTWDCAFAIQYFFVAGLAERPEFYNTIVSAYKF

|.||| ||||.:||||||||.|||||.|.||..|.:||||:||||: .|: : |:

354 FKRLQERFKDVIFHGPQGMTTMGTNGTQVWDTSFCLQYFFMAGLADLDEYEELII.....

421 LCHAQFDTECVPGSYRDKRKGAWGFSTKTQGYTVADCTAEAIKAIIMVKNSPVFSEVHHM

|||| |.:.||||.|||||.||||||||||:|||| | |. : .

409 ...............RDKRIGCFPFSTKEQGYTVSDCTAEAIKAILMVKNHPKFAYLGDY

481 ISSERLFEGIDVLLNLQNIGSFEYGSFATYEKIKAPLAMETLNPAEVFGNIMVEYPYVEC

|..: | .|||.||.|||:||:.:|||.|||..:| |:|.:||||||||||||||||||

454 IDEDLLKKGIDGLLSLQNVGSYHFGSFSTYESTRANPALEKINPAEVFGNIMVEYPYVEC

541 TDSSVLGLTYFHKYFDYRKEEIRTRIRIAIEFIKKSQLPDGSWYGSWGICFTYAGMFALE

|||||||||||:. :|||:.:| | | ::.|| ..| .||||||.||:|||||||||||

514 TDSSVLGLTYFREHWDYRRHDIDTAIERGVKFICDAQQEDGSWYGCWGVCFTYAGMFALE

601 ALHTVGETYENSSTVRKGCDFLVSKQMKDGGWGESMKSSELHSYVDSEKSLVVQTAWALI

|| .|.: ||....||||||||||||| ||||:||:||.| |.|| :.:::||||.|.||

574 ALASVNQYYETNEVVRKGCDFLVSKQMADGGWSESIKSCETHTYVRGKRGMVVQTSWVLI

661 ALLFAEYPNKEVIDRGIDLLKNRQEESGEWKFESVEGVFNHSCAIEYPSYRFLFPIKALG

:|::|.||:|||||||::|:..||...|::.||.|||:|||||:||||.|:|||||||||

634 GLILAKYPHKEVIDRGVQLIMSRQKTRGDFDFEAVEGIFNHSCGIEYPNYKFLFPIKALG

721 MYSRAYETHTL

:||:.||

694 LYSKEYEQ

# Fig. S4 The protein sequences comparison between heterologous lanosterol synthases and ERG7.


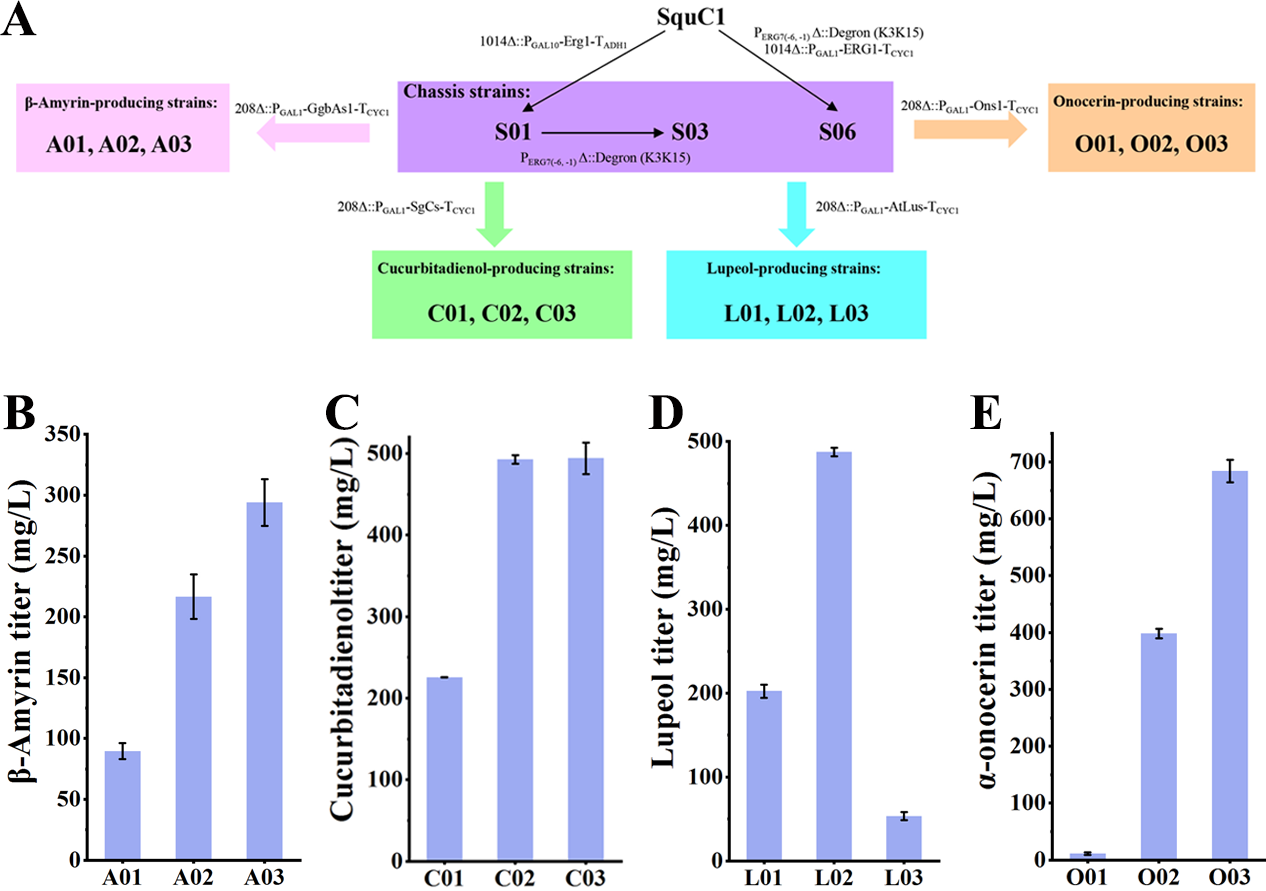


# Fig. S5 Removing the bottleneck to stimulate PTs production. The construction diagram of PT-producing strains (A). The titers of β-amyrin (B), cucurbitadienol (C), lupeol (D) and α-onocerin (E).


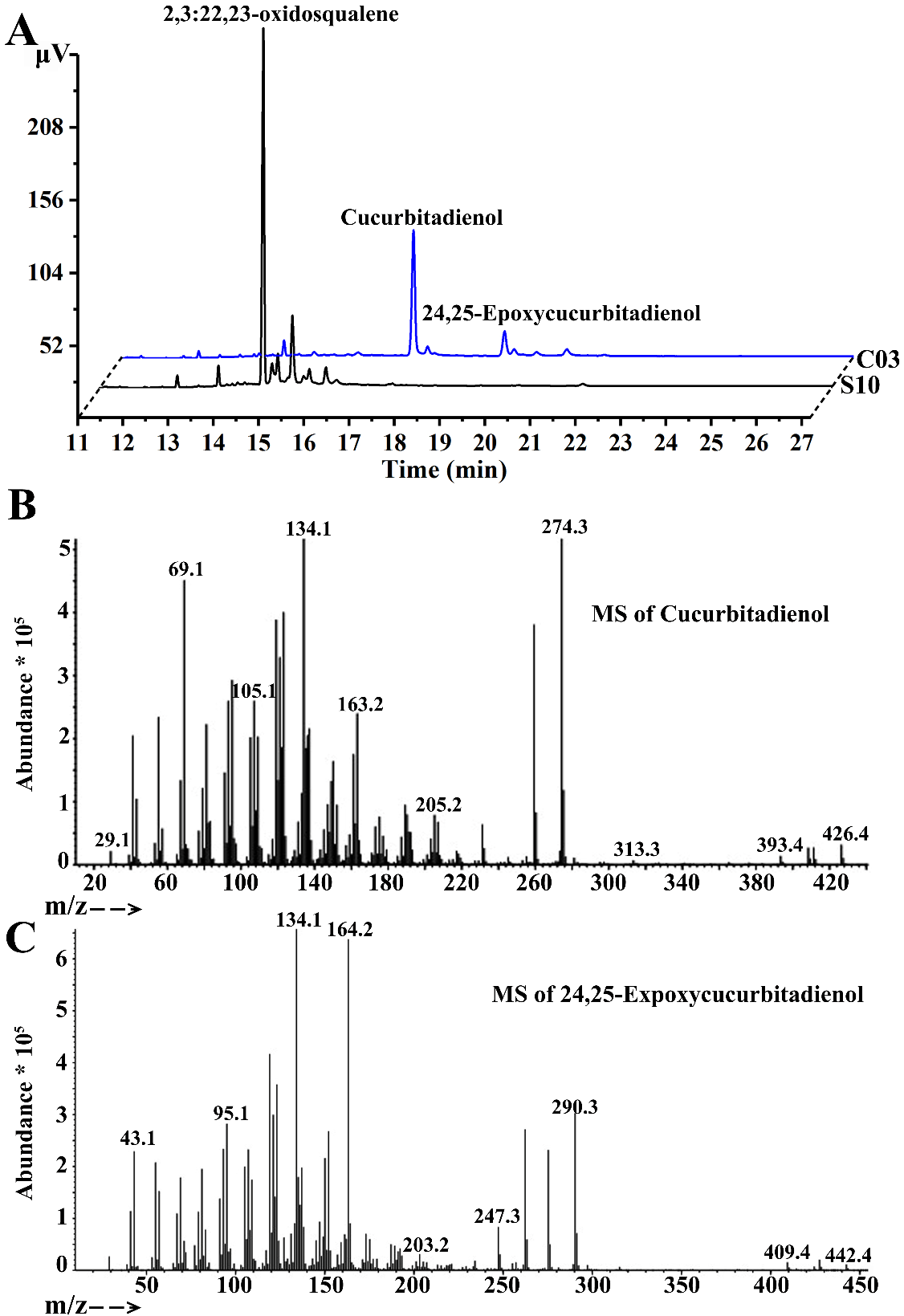


# Fig. S6 Detection of 24,25-epoxycucurbitadienol. (A) GC chromatograms of strains S10 and C03. (B) GC-MS analysis of cucurbitadienol. (C) GC-MS analysis of 24,25-epoxycucurbitadienol. The GC detection conditions for epoxycucurbitadienol are follows: The GC system (Agilent 7820 A, USA) was equipped with an HP-5 capillary column (30 m × 0.25 mm, 0.25 μm film thickness) and a flame ionization detector (FID) using N2 (1 mL/min) as the carrier gas. The oven temperature was programmed to rise from an initial temperature of 200 °C for 2 min, gradually increased to 280 °C at a rate of 20 °C/min, maintained for 2 min, gradually increased to 305 °C at a rate of 10 °C/min, maintained for 14 min.


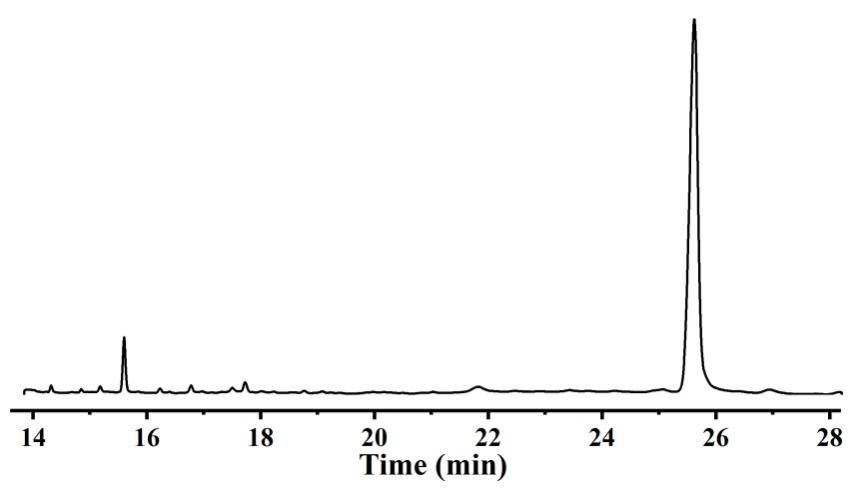


# Fig. S7 Detection of the white solids accumulated on the tank wall during the fed-batch fermentation.


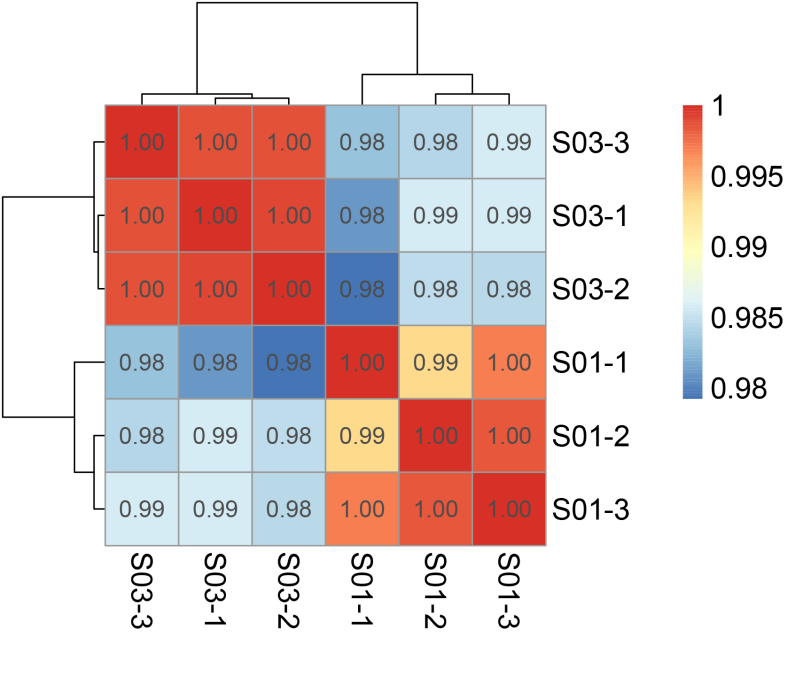


# Fig. S8 Correlation between samples. The correlation coefficient is highly correlated between 0.8 and 1 and is low correlated less than 0.8.

# Supplementary Tables

# Table S1. *S. cerevisiae* strains used in this work.

| **Strains** | **Host strains** | | **Descriptions** | **Resources** |
| --- | --- | --- | --- | --- |
| SquC1 |  | gal80Δ::T_ADH1_-Erg20-P_GAL10_-P_GAL1_-Erg9-T_CYC1_;  gal1-7Δ::T_ADH1_-P_GAL4_-Gal4-T_CYC1_;  gre3Δ::T_ADH1_-Idi1-P_GAL10_-P_GAL1_-tHmg1-T_CYC1_ | | In our lab [1] |
| S01 | SquC1 | 1014Δ::T_ADH1_-Erg1-P_GAL10_-P_GAL1_-T_CYC1_ | | This study |
| S02 | S01 | P_ERG7(-6, -1)_ Δ::Degron (K15) | | This study |
| S03 | S01 | P_ERG7(-6, -1)_ Δ::Degron (K3K15) | | This study |
| S04 | S01 | P_ERG7(-6, -1)_ Δ::Degron (KN113) | | This study |
| S05 | S01 | P_ERG7(-6, -1)_ Δ::Degron (KN119) | | This study |
| S01G | SquC1 | 1014Δ::T_ADH1_-Gfp-Erg1-P_GAL10_-P_GAL1_-T_CYC1_ | | This study |
| S03G | S01G | P_ERG7(-6, -1)_ Δ::Degron (K3K15) | | This study |
| S01GR | S01G | Erg7_(2194, 2217)_ Δ:: mCherry | | This study |
| S02GR | S02 | 1014Δ::T_ADH1_-Gfp-Erg1-P_GAL10_-P_GAL1_-T_CYC1_  Erg7_(2194, 2217)_ Δ:: mCherry | | This study |
| S03GR | S03G | Erg7_(2194, 2217)_ Δ:: mCherry | | This study |
| S03Gg | S01 | Erg7Δ::GgLss1 | | This study |
| S03Tf | S01 | Erg7Δ::TfLss1 | | This study |
| S03Pb | S01 | Erg7Δ::PbLss1 | | This study |
| S03GgG | S01G | Erg7Δ::GgLss1 | | This study |
| S06 | SquC1 | P_ERG7(-6, -1)_ Δ::Degron (K3K15)  1014Δ::T_ADH1_- P_GAL10_-P_GAL1_-ERG1-T_CYC1_ | | This study |
| A01 | S01 | 208Δ::T_ADH1_-P_GAL10_-P_GAL1_-GgbAs1-T_CYC1_ | | This study |
| A02 | S03 | 208Δ::T_ADH1_-P_GAL10_-P_GAL1_-GgbAs1-T_CYC1_ | | This study |
| A03 | S06 | 208Δ::T_ADH1_-P_GAL10_-P_GAL1_-GgbAs1-T_CYC1_ | | This study |
| L01 | S01 | 208Δ::T_ADH1_-P_GAL10_-P_GAL1_-AtLus1-T_CYC1_ | | This study |
| L02 | S03 | 208Δ::T_ADH1_-P_GAL10_-P_GAL1_-AtLus1-T_CYC1_ | | This study |
| L03 | S06 | 208Δ::T_ADH1_-P_GAL10_-P_GAL1_-AtLus1-T_CYC1_ | | This study |
| C01 | S01 | 208Δ::T_ADH1_-P_GAL10_-P_GAL1_-SgCs-T_CYC1_ | | This study |
| C02 | S03 | 208Δ::T_ADH1_-P_GAL10_-P_GAL1_-SgCs-T_CYC1_ | | This study |
| C03 | S06 | 208Δ::T_ADH1_-P_GAL10_-P_GAL1_-SgCs-T_CYC1_ | | This study |
| O01 | S01 | 208Δ::T_ADH1_-P_GAL10_-P_GAL1_-Ons1-T_CYC1_ | | This study |
| O02 | S03 | 208Δ::T_ADH1_-P_GAL10_-P_GAL1_-Ons1-T_CYC1_ | | This study |
| O03 | S06 | 208Δ::T_ADH1_-P_GAL10_-P_GAL1_-Ons1-T_CYC1_ | | This study |
| AC01 | A01 | 1021Δ::T_ADH1_-Erg10-P_GAL10_-P_GAL1_-ACS1-T_CYC1_  δ15Δ::T_ADH1_-Erg12-P_GAL10_-P_GAL1_-Erg13-T_CYC1_  1622Δ::T_ADH1_-Erg19-P_GAL10_-P_GAL1_-Erg8-T_CYC1_  lpp1Δ:: T_ADH1_-Idi1-P_GAL10_-P_GAL1_-tHmg1-T_CYC1_  dpp1Δ:: T_ADH1_-Erg20-P_GAL10_-P_GAL1_-Erg9-T_CYC1_ | | This study |
| AC20 | AC01 | 308Δ::T_ADH1_-GgbAs1-P_GAL10_-P_GAL1_-Erg1-T_CYC1_  P_ERG7(-6, -1)_ Δ::Degron (K15) | | This study |
| AC21 | AC01 | 308Δ::T_ADH1_-GgbAs1-P_GAL10_-P_GAL1_-Erg1-T_CYC1_  P_ERG7(-6, -1)_ Δ::Degron (K3K15) | | This study |
| AC22 | AC01 | 308Δ::T_ADH1_-Erg1-P_GAL10_-P_GAL1_-GgbAs1-T_CYC1_  P_ERG7(-6, -1)_ Δ::Degron (K15) | | This study |
| AC23 | AC01 | 308Δ::T_ADH1_-Erg1-P_GAL10_-P_GAL1_-GgbAs1-T_CYC1_  P_ERG7(-6, -1)_ Δ::Degron (K3K15) | | This study |
| CGFP01 | SquC1 | 1014Δ:: P_GAL1_-Gfp-T_CYC1_ | | This study |
| CGFP02 | CGFP01 | 911Δ:: P_GAL80_- Gal80-T_CYC1_ | | This study |
| CGFP03 | CGFP01 | 911Δ:: P_GAL80_-K15-Gal80-T_CYC1_ | | This study |
| CGFP04 | CGFP01 | 911Δ:: P_GAL80_-K3K15-Gal80-T_CYC1_ | | This study |
| CGFP05 | CGFP01 | 911Δ:: P_GAL80_-KN119-Gal80-T_CYC1_ | | This study |
| CGFP06 | CGFP01 | 911Δ:: P_GAL80_-KN113-Gal80-T_CYC1_ | | This study |
| AC24 | AC22 | 911Δ:: P_GAL80_-KN113-Gal80-T_CYC1_ | | This study |
| AC25 | AC24 | his3::P_URA3_-Ura3-T_ADH1_-P_Trp1_-Trp1-T_CYC1_-P_HIS3_-His3-T_PGK1-_P_Leu2_-Leu2-T_TPS1_ | | This study |

# Table S2. Plasmids used in this study.

| **Name** | **Description** | **Source** |
| --- | --- | --- |
| pZT20 | Cloning vector | In our laboratory |
| pZT20-Erg1-10 | T_ADH1_-Erg1-P_GAL10_-P_GAL1_-T_CYC1_ | This study |
| pZT20-Erg1-1 | T_ADH1_- P_GAL10_-P_GAL1_- Erg1-T_CYC1_ | This study |
| pZT20-bAs1-1 | T_ADH1_- P_GAL10_-P_GAL1_- bAs1-T_CYC1_ | This study |
| pZT20-bAs1-10 | T_ADH1_- bAs1-P_GAL10_-P_GAL1_- T_CYC1_ | This study |
| pZT20-AtLus1 | T_ADH1_- P_GAL10_-P_GAL1_- AtLus1-T_CYC1_ | This study |
| pZT20-SgCs | T_ADH1_- P_GAL10_-P_GAL1_- SgCs-T_CYC1_ | This study |
| pZT20-Ons1 | T_ADH1_- P_GAL10_-P_GAL1_- Ons1-T_CYC1_ | This study |
| pZT20-Erg10-Acs1 | T_ADH1_-Erg10-P_GAL10_-P_GAL1_-Acs1-T_CYC1_ | This study |
| pZT20-Erg13-Erg12 | T_ADH1_-Erg12-P_GAL10_-P_GAL1_-Erg13-T_CYC1_ | This study |
| pZT20-Erg8-Erg19 | T_ADH1_-Erg19-P_GAL10_-P_GAL1_-Erg8-T_CYC1_ | This study |
| pZT20-tHmg1-IDI1 | T_ADH1_-IDI1-P_GAL10_-P_GAL1_-tHmg1-T_CYC1_ | This study |
| pZT-Erg9-Erg20 | T_ADH1_-Erg20-P_GAL10_-P_GAL1_-Erg9-T_CYC1_ | This study |
| pSCM-1014 | gRNA plasmid targeting to 1014 site | This study |
| pSCM-PERG7 | gRNA plasmid targeting to PERG7 site | This study |
| pSCM-PERG11 | gRNA plasmid targeting to PERG11 site | This study |
| pSCM-ERG7C | gRNA plasmid targeting to ERG7C site | This study |
| pSCM-tERG7 | gRNA plasmid targeting to tERG7 site |  |
| pSCM-208 | gRNA plasmid targeting to 208 site | This study |
| pSCM-1021 | gRNA plasmid targeting to 1021 site | This study |
| pSCM-δ15 | gRNA plasmid targeting to δ15 site | This study |
| pSCM-1622 | gRNA plasmid targeting to 1622 site | This study |
| pSCM-lpp1 | gRNA plasmid targeting to lpp1 site | This study |
| pSCM-dpp1 | gRNA plasmid targeting to dpp1 site | This study |
| pSCM-308 | gRNA plasmid targeting to 308 site | This study |
| pSCM-911 | gRNA plasmid targeting to 911 site | This study |
| pSCM-his3 | gRNA plasmid targeting to his3 site | This study |

# Table S3. Primers used in this study.

| **primer names** | **5'-3'sequences** |
| --- | --- |
| **Primers used for gene expression plasmid construction:** | |
| reBamHI-ERG1-F | GGAGAAAAAACcccggatccATGTCTGCTGTTAACGTTGC |
| reKpnI-ERG1-R | gctagccgcggtaccTTATTAACCAATCAACTCACCAAACAAAAA |
| pGaL10-ERG1-F | ATAAGAATGCGGCCGCATGTCTGCTGTTAACGTTGC |
| tADH1-ERG1-R | GAAGATCTTTAACCAATCAACTCACCAAACAAA |
| ERG1-GFP-R | CAGGTGCAACGTTAACAGCAGACATtttgtatagttcatccatgccatgt |
| pGal1-GFP-F | GTCAAGGAGAAAAAACcccggatccatgagtaaaggagaagaacttttca |
| pGaL1-GgbAS-F | AGGAGAAAAAACCCCGGATCCATGTGGAGACTTAAAATCGCTG |
| tCYC1-GgbAS-R | GCGGTACCAAGCTTACTCGAGTCATGTAAGGCAAACTGGTGT |
| re-gal10-bAS1-F | CGAATTCAACCCTCACTATGTGGAGACTTAAAATCGCTG |
| re-TADH1-bAs1-R | CCTCTGGCGAAGAATTGTCATGTAAGGCAAACTGGTG |
| reBamHI-AtLus1-F | aggagaaaaaaccccgATGTGGAAGTTGAAGATCGG |
| reKpnI-AtLus1-R | atcttagctagccgcgTTAATTAACAATAAAAACAACTTTTCTATATTC |
| reBamHI-optSgCS-F | aggagaaaaaaccccgATGTGGAGATTGAAAGTTGG |
| KpnI-optSgCS-R | atcttagctagccgcgTTATTCTGTTAAAACTCTATGACAAT |
| reBamHI-ons1-F | ggagaaaaaaccccggatccATGTGGAAATTAAAAACAGCCG |
| KpnI-ons1-R | tcttagctagccgcggtaccTTATTCTTGATGAAACAAAGGTG |
| GAL1-ACS1-F | GTCAAGGAGAAAAAACCCCggatccATGTCGCCCTCTGCCGTACA |
| CYC1-ACS1-R | gcggtaccaagcttactcgagTTACAACTTGACCGAATCAATTAG |
| GAL10-ERG10-R | caaccctcactaaagggcggccgcatgtctcagaacgtttacattg |
| ADH1-ERG10-F | CTTGTAATCCATCGATACTAGTtcatatcttttcaatgacaatagag |
| GAL10-ERG12-R | TCACTAAAGGGCGGCCGCatgtcattaccgttcttaacttct |
| ADH1-ERG12-F | CTTGTAATCCATCGATACTAGTttatgaagtccatggtaaattcg |
| GAL1-ERG13-F | GGAGAAAAAACCCCGGATCCatgaaactctcaactaaactttgtt |
| CYC1-ERG13-R | AACTTCTGTTCCATGTCGACttattttttaacatcgtaagatcttc |
| GAL1-ERG8-F | GGAGAAAAAACCCCGGATCCatgtcagagttgagagcctt |
| CYC1-ERG8-R | AACTTCTGTTCCATGTCGACttatttatcaagataagtttccgg |
| GAL10-ERG19-R | TCACTAAAGGGCGGCCGCatgaccgtttacacagcatc |
| ADH1-ERG19-F | CTTGTAATCCATCGATACTAGTttattcctttggtagaccagtct |
| GAL1-tHmg1-F | CTAAAGGGCGGCCGCATGGACCAATTGGTGAAAACTGA |
| CYC1-tHmg1-R | CCTTAATTAATTAGGATTTAATGCAGGTGACGG |
| GAL10-IDI1-F | AACCCCGGATCCatgactgccgacaacaatag |
| ADH1-IDI1-R | CTTACTCGAGttatagcattctatgaatttgcctg |
| GAL1-ERG9-F | AGAAAAAACCCCGGATCCATGGGAAAGCTATTACAATTGGCA |
| CYC1-ERG9-R | TCTGTTCCATGTCGACTCACGCTCTGTGTAAAGTGTATAT |
| GAL10-ERG20-F | TCACTAAAGGGCGGCCGCatggcttcagaaaaagaaattagg |
| ADH1-ERG20-R | CTTGTAATCCATCGATACTAGTttatttacttctcttgtaaaccttg |
| PGAL10-GFP-F | CCTCACTAAAGGGCGGCCGCatgagtaaaggagaagaacttttcac |
| ERG1-GFP-R | GCAACGTTAACAGCAGACATtttgtatagttcatccatgccatg |
| **Primers used for gRNA plasmid construction:** | |
| gRNA-Erg7C-F | AGTGGATGGTGGGTCGTTTGgtttAagagctaTGCTGgaaaCAG |
| gRNA-Erg7C-R | CAAACGACCCACCATCCACTgatcatttatctttcactgcggag |
| gRNA-tErg7-F | TTATTTATAAGTGTTGTTAGgtttAagagctaTGCTGgaaaCAGC |
| gRNA-tErg7-R | CTAACAACACTTATAAATAAgatcatttatctttcactgcggag |
| gRNA-DPP1-F | CGATTCAGATGTCACCCTGGGTTTAAGAGCTATGCTGGAAACAG |
| gRNA-DPP1-R | CCAGGGTGACATCTGAATCGGATCATTTATCTTTCACTGCGGAG |
| gRNA-LPP1-F | AACTCCTACATCAACGCCTAGTTTAAGAGCTATGCTGGAAAC |
| gRNA-LPP1-R | TAGGCGTTGATGTAGGAGTTGATCATTTATCTTTCACTGCGGA |
| gRNA-308-F | TAAGCGGAAGGGGAAGGGTTGTTTAAGAGCTATGCTGGAAACAG |
| gRNA-308-R | AACCCTTCCCCTTCCGCTTAGATCATTTATCTTTCACTGCGGAG |
| gRNA-911-F | GTAATATTGTCTTGTTTCCCGTTTAAGAGCTATGCTGGAAACAG |
| gRNA-911-R | GGGAAACAAGACAATATTACGATCATTTATCTTTCACTGCGGAG |
| gRNA-1014-F | CATATTCACGGGATACTAGGGTTTAAGAGCTATGCTGGAAACAG |
| gRNA-1014-R | CCTAGTATCCCGTGAATATGGATCATTTATCTTTCACTGCGGAG |
| gRNA-δ15-F | AAATCCGAACAACAGAGCATGTTTAAGAGCTATGCTGGAAACAG |
| gRNA-δ15-R | ATGCTCTGTTGTTCGGATTTGATCATTTATCTTTCACTGCGGAG |
| gRNA-his3-F | ACATACTTACTGACATTCATGTTTAAGAGCTATGCTGGAAAC |
| gRNA-his3-R | ATGAATGTCAGTAAGTATGTGATCATTTATCTTTCACTGCGG |
| gRNA-1622-F | CTTCCATATCCGCCGAATGCgtttAagagctaTGCTGgaaaCAG |
| gRNA-1622-R | GCATTCGGCGGATATGGAAGgatcatttatctttcactgcggag |
| gRNA-1021-F | AATTACCACCACACAGAGGCgtttAagagctaTGCTGgaaaCAG |
| gRNA-1021-R | GCCTCTGTGTGGTGGTAATTgatcatttatctttcactgcggag |
| gRNA-208-F | AGCCAGTAACAAGCTTGTGTgtttAagagctaTGCTGgaaaCAG |
| gRNA-208-R | ACACAAGCTTGTTACTGGCTgatcatttatctttcactgcggag |
| gRNA-PERG7-F | CTGTTTTGTACTTTCTTTGTgtttAagagctaTGCTGgaaaCAG |
| gRNA-PERG7-R | ACAAAGAAAGTACAAAACAGgatcatttatctttcactgcggag |
| gRNA-PERG11-F | CAGAACAAACGAGTAATACAgtttAagagctaTGCTGgaaaCAG |
| gRNA-PERG11-R | TGTATTACTCGTTTGTTCTGgatcatttatctttcactgcggag |
| **Primers used for the construction of engineered yeasts:** | |
| tErg7:: |  |
| ERG7-mCherry-F | GGTGGTGGTTGTGGTatggtgagcaagggcgagga |
| tERG7-mCherry-R | GTTTCTAATTGTTGCAGCCTCTTTActtgtacagctcgtccatgc |
| mCherry-ERG7-R | tcgcccttgctcaccatACCACAACCACCACCAAGCGTATGTGTTTCATATGCCC |
| ERG7-F | TACGTACACGCATCAGAATCG |
| mCherry-tERG7-F | cggcatggacgagctgtacaagTAAAGAGGCTGCAACAATTAGAAACT |
| tERD7-DR | CTATTGGCCTGGTACCAAACC |
| 208:: |  |
| TADH1-208-DF | ATAGCATGAGGTCGCTCCAATTCAGAGGAGTTTGTCTGAAAACCATC |
| 208D-TADH1-R | GAAGATGGTTTTCAGACAAACTCCTCTGAATTGGAGCGACCTCAT |
| TCYC1-208-UF | TGAGAAGGTTTTGGGACGCTCGAAGTTCAGTCCGAAAACAGCTCC |
| 208U-TCYC1-R | TGAGAAGGTTTTGGGACGCTCGAAGTTCAGTCCGAAAACAGCTCC |
| cen208-UF | GTCTAGTCTATCAGCCTCCG |
| cen208-DR | CGAAGAAGGCATGGGAATCT |
| 308:: |  |
| ARS308-UR | TCTCACCGCATGACAAGTGG |
| tADH1-308U-F | ATAGCATGAGGTCGCTCCAATTCAGCATAAACATGGCATGGCGATCA |
| 308U-tADH1-R | CGCTGATCGCCATGCCATGTTTATGCTGAATTGGAGCGACCTCAT |
| 308D-TCYC1-R | AGTTTCAATAGCTTGCAGCGTAGCTCTTCGAGCGTCCCAAAACCT |
| TCYC1-308-DR | TGAGAAGGTTTTGGGACGCTCGAAGAGCTACGCTGCAAGCTATTG |
| ARS308-DR | ACGGATGACACAGTGGttag |
| DPP1:: |  |
| DPP1-UF | TCTGTGGCTGCTTATCCCAGC |
| DPP1-TADH1-UR | GCATGAGGTCGCTCCAATTCAGCCTTTGTCCGATGAAGGTATGTAA |
| DPP1-TADH1-R | TTACATACCTTCATCGGACAAAGGCTGAATTGGAGCGACCTCATGC |
| DPP1-TCYC1-R | CGACCAAAATGAACAGAGTTTCGCTTCGAGCGTCCCAAAACCT |
| DPP1-TCYC1-DF | AGGTTTTGGGACGCTCGAAGCGAAACTCTGTTCATTTTGGTCG |
| DPP1-DR | CTGTCTTGTGATCGCATACTCTGC |
| LPP1:: |  |
| LPP1-UF | TGACCTTCACCGACGGATTC |
| LPP1-TADH1-UR | GCATGAGGTCGCTCCAATTCAGCCTTCACCGGTTAGTGTTTAGTAA |
| LPP1-TADH1-R | TTACTAAACACTAACCGGTGAAGGCTGAATTGGAGCGACCTCATGC |
| LPP1-TCYC1-R | ATTCTACCAAGGATGATCTCTGTCCTTCGAGCGTCCCAAAACCT |
| LPP1-TCYC1-DF | AGGTTTTGGGACGCTCGAAGGACAGAGATCATCCTTGGTAGAAT |
| LPP1-DR | CTCGACAACTTTCAGATTGCTG |
| 1622:: |  |
| ARS1622-UF | CCACCAATTGCAGAGGGTAG |
| tADH1-1622-UR | ATAGCATGAGGTCGCTCCAATTCAGCCGTAAGTTCTTGGAAATCACT |
| 1622U-tADH1-R | CCTAGTGATTTCCAAGAACTTACGGCTGAATTGGAGCGACCTCAT |
| 1622D-tCYC1-R | CTCCGAATCATTGTTCGGGACTTCGAGCGTCCCAAAACCTTC |
| tCYC1-1622-DF | GAAGGTTTTGGGACGCTCGAAGTCCCGAACAATGATTCGGAG |
| ARS1622-DR | GTCATCAGGTGTAATGGAAGCT |
| δ15:: |  |
| δ15-UF | GGAAGCTGAAATGCAAAGATCG |
| tADH1-δ15-UR | ATAGCATGAGGTCGCTCCAATTCAGCGCAAACAAACTTAAATATATGC |
| δ15U-tADH1-R | TAGCATATATTTAAGTTTGTTTGCGCTGAATTGGAGCGACCTCAT |
| δ15D-tCYC1-R | AAGAAAAACTAACACATTAATGTAGCTTCGAGCGTCCCAAAACCT |
| tCYC1-δ15D-F | TGAGAAGGTTTTGGGACGCTCGAAGCTACATTAATGTGTTAGTTTTTCTTT |
| δ15-DR | CCGATAACGCCAGGCGCCTT |
| ARS1021:: |  |
| ARS1021-UF | CTCCAAGGTAGGAAAGTCGA |
| 1021D-tCYC1-R | ACAGAATGAATTTCATCACGTGCGTcttcgagcgtcccaaaacct |
| tCYC1-ARS1021-DF | tgagaaggttttgggacgctcgaagACGCACGTGATGAAATTCAT |
| 1021UHA-TADH1-R | TCCAGTGTCTCTTAGCAGTTAAACCCTGAATTGGAGCGACCTCAT |
| TADH1-1021-UR | ATGAGGTCGCTCCAATTCAGGGTTTAACTGCTAAGAGACACTG |
| ARS1021-DR | CTATGAATTTGTCAGCCGCA |
| PERG7:: |  |
| ERG7-DHA-R | TTCAAAGTAGTAGAATGGGGGTAA |
| ub-pERG72-UR | TCAAAGTCTTGACGAAAATCTGCATTGTACTTTCTTTGTGGGCGA |
| pERG72U-ub-F | AATCGTCGCCCACAAAGAAAGTACAATGCAGATTTTCGTCAAGAC |
| ERG7D-K15-R | CGATTGTGTCAGAATAAAATTCTGTCATACCAGAACCCTTAACCAAAG |
| K15-ERG7-DF | TCTTTGGTTAAGGGTTCTGGTATGACAGAATTTTATTCTGACACAATCG |
| ERG7C:: |  |
| GgLSS-ERG7-UR | TTCTAACAACACCATCTGCAGCCATCTGTTTTGTACTTTCTTTGTGGGC |
| ERG7U-GgLSS-F | CGCCCACAAAGAAAGTACAAAACAGATGGCTGCAGATGGTGTTGT |
| ERG7D-GgLSS-R | CTCTAACAACACTTATAAATAAAACTTAATCTGATAAAGCTGTTTTTGGAG |
| GgLSS-ERG7-DF | TCCAAAAACAGCTTTATCAGATTAAGTTTTATTTATAAGTGTTGTTAGAGGCTGC |
| PbLSS1-pERG7-UR | TCTGAATAATACACTGTCATCTGTTTTGTACTTTCTTTGTGGG |
| pERG7U-PbLSS1-F | ACAAAGAAAGTACAAAACAGATGACAGTGTATTATTCAGAGAAAATCG |
| tERG7-PbLSS1-R | ACAACACTTATAAATAAAACTTATTGCTCATACTCTTTGGAATATAACC |
| PbLSS1-tERG7-DF | CCAAAGAGTATGAGCAATAAGTTTTATTTATAAGTGTTGTTAGAGGC |
| TfLSS1-pERG7-UR | GCCGGATTTTGCTTTCTTTCGCCATCTGTTTTGTACTTTCTTTGTGGG |
| pERG7U-TfLSS1-F | CGCCCACAAAGAAAGTACAAAACAGATGGCGAAAGAAAGCAAAATCC |
| tERG7D-TfLSS1-R | GCAGCCTCTAACAACACTTATAAATAAAACTCAGAACAAAGCCTCATTGCC |
| TfLSS1-tERG7-DF | TGGCAATGAGGCTTTGTTCTGAGTTTTATTTATAAGTGTTGTTAGAGG |
| 1014:: |  |
| ARS1014-UF | GTGAAGCAATTAGACGCAAACG |
| TADH1-ARS1014-UR | ATAGCATGAGGTCGCTCCAATTCAGCATACTACGAGTTCTCCTCG |
| ARS1014U-TADH1-R | GTTCTCGAGGAGAACTCGTAGTATGCTGAATTGGAGCGACCTCAT |
| ARS1014D-tCYC1-R | CATGAAATTTGTAACATGCTGACCTCTTCGAGCGTCCCAAAACCT |
| tCYC1-ARS1014-DF | TGAGAAGGTTTTGGGACGCTCGAAGAGGTCAGCATGTTACAAATTTC |
| ARS1014-DR | GGCATGCAATGTATATGGCT |
| PERG11:: |  |
| ERG11-R | GGAACGTAGCTCTTGAAGGC |
| K3K15-ERG11-DF | GGTTTCTTTGGTTAAGGGTTCTGGTATGTCTGCTACCAAGTCAATCG |
| ERG11D-K3K15-R | CCAACGATTGACTTGGTAGCAGACATACCAGAACCCTTAACCAAAG |
| pERG11-ub-F | CTTGAATAGAAACAGAACAAACGAGATGCAGATTTTCGTCAAGACTTT |
| ub-pERG11-UR | TCAAAGTCTTGACGAAAATCTGCATCTCGTTTGTTCTGTTTCTATTCAAG |
| PERG11-F | AGTCTCTTGCAGAACACAAT |
| 911:: |  |
| ARS911-UF | TAGTGGAGGCAAGGTTGCAT |
| TADH1-ARS911-UR | ATAGCATGAGGTCGCTCCAATTCAGttatGCCCATTCAACATccg |
| ARS911U-TADH1-R | aaaatcggATGTTGAATGGGCataaCTGAATTGGAGCGACCTCAT |
| ARS911D-tCYC1-R | tcatttttcatttacttCTCCAGGGCTTCGAGCGTCCCAAAACCT |
| tCYC1-ARS911-DF | TGAGAAGGTTTTGGGACGCTCGAAGCCCTGGAGaagtaaatgaaa |
| ARS911-DR | CGGAAACATTATACTCAAGTCGC |
| His3:: |  |
| CHiS3-UF | CACGGCATTAGTCAGGGAAGTC |
| His3-TADH1-UR | GCATGAGGTCGCTCCAATTCAGGCTTGACCGAGAGCAATCCC |
| His3-TADH1-F | GGGATTGCTCTCGGTCAAGCCTGAATTGGAGCGACCTCATGC |
| CYC1-TPGK1-R | CGAAAATTCTGCGTTCGTTACTTCGAGCGTCCCAAAACCT |
| CYC1-TPGK1-F | AGGTTTTGGGACGCTCGAAGTAACGAACGCAGAATTTTCG |
| TTPS1-His3-R | CTGTTCGTATACATACTTACTGACCGGTGTGAACAGCGACAATA |
| TTPS1-His3-UF | TATTGTCGCTGTTCACACCGGTCAGTAAGTATGTATACGAACAG |
| CHiS3-DR | CCAGAGGCGTCCACTGGAAT |

# Table S4. Heterologous lanosterol synthase sequences:

| **Gene names** | **5'-3'sequences** |
| --- | --- |
| GgLss1 | ATGGCTGCAGATGGTGTTGTTAGAAGAAGAGGTGGTCCATGGAGAACTGCTGCAGCTACAGCTTTGCCATCTTGGAGATTAAGATGTGAAGGTGGTAGACAAAGATGGTGTTATTTGGATGATGGTGTTGAAGATGAAGAAGCAGCTGGTGGTAGAAGAGCACAAACTGCTTTGGAAGCACATTCTGTTGGTTTGGATACTGATGCTTTGTTGAGAACATTACCAGCAGCTGGTACTGCAAGAGAAGCAGCTAGAAACGGTATGAGATTCTACGCTACTTTGCAAGCAGAAGATGGTCATTGGGCTGGTGACTACGGTGGTCCATTATTTTTGTTGCCAGGTTTGTTGATCGTTTGTCATACAGCAAGAATTCCATTGCCAGATGGTTTTAGAAGAGAAATGGTTAGATATTTGAGATCTGTTCAATTACCAGATGGTGGTTGGGGTTTGCATGTTGAGGATAAGTCAACTGTTTTCGGTACAGCTTTGAACTACGTTGCATTGAGAATCTTGGGTTTGGGTCCAGATGATCCAGATATCGTTAGAGCAAGAGTTAATTTGCATTCTAAAGGTGGTGCTGTTGGTATTCCATCATGGGGTAAATTCTGGTTGGCTGTTTTGAACGTTTATTCTTGGGAGGGTATGAATACTTTGTTACCAGAAATGTGGTTGTTACCAACATGGTTTCCAGCTCATCCATCAAGATTGTGGTGTCATTGTAGACAAGTTTACTTACCAATGTCTTACTGTTACGCTAAGAGATTGTCAGCAGAAGAAGATGAATTGATCAGATCTTTGCAACAAGAATTGTACGTTCAAGATTACGCTTCAATTGATTGGCCAGCACAAAGAAATAATGTTGCAGCTTGTGATGTTTATACTCCACATTCTTGGTTGTTGGGTATCGCATACGCTATCATGAACGTTTACGAAGCACATCATTCAACTTATTTGAGACAAAGAGCTATCACAGAATTGTACGATCATATCAAGGCAGATGATAGATTCACTAAGTGTATCTCTATCGGTCCAATCTCAAAGACAATTAATATGTTGGTTAGATGGTTCGTTGATGGTGAAAATTCTCCAGCTTTTCAAGAACATGTTTCAAGAATCCCAGATTATTTGTGGTTAGGTTTGGATGGTATGAAAATGCAAGGTACTAATGGTTCTCAATTGTGGGATACAGCATTTGCTGTTCAAGCATTTTTGGAAGCAGAAGCTCAAAAGATTCCAGAATTCATGTCTTGTTTGCAAAACGCACATGAATTCTTGAGATTCACTCAAATCCCAGAAAATCCACCAGATTACCAAAAGTACTACAGACATATGAATAAGGGTGGTTTTCCATTTTCTACTAGAGATTGTGGTTGGATTGTTGCTGATTGTACAGCAGAAGGTTTGAAGTCAATCATGTTGTTGCAAGAAAAGTGTCCTTTTATTGCTAACCCAGTTCCAGCAGAAAGATTATTTGATGCTGTTAACGTTTTGTTGTCTATGAAGAACTCAGATGGTGGTTTCGCAACTTATGAAACAAAAAGAGGTGGTCATTTGTTGGAATTGTTGAACCCATCTGAAGTTTTCGGTGACATCATGATCGATTACACTTACGTTGAATGTACATCAGCTGTTATGCAAGCATTGAGACATTTTCAAGATGTTTACCCAGAACATAGAGCTCCAGAAATTAGAGAAACTTTGCAAAAGGGTTTGGATTTCTGTAGAAAGAAACAACAAGCTGATGGTTCTTGGGAAGGTTCATGGGGTGTTTGTTTTACTTATGGTACATGGTTTGGTTTAGAAGCATTTGCTTCTATGCAACATGTTTACAGAGATGGTGTTGCTTGTAGAGAAGTTGCAAGAGCTTGTCAATTCTTGTTGTCAAAGCAAATGACTGATGGTGGTTGGGGTGAAGATTTCGAATCTTGTGAACAAAGAACATACGTTCAATCTTCAACTTCACAAATCCATAACACATGTTGGGCTTTGTTAGGTTTGATGGCAGTTAGATACCCAGATACAGGTGTTTTGGAAAGAGGTATTAAATTGTTGATCGATAAGCAATTACCAAATGGTGACTGGCCACAAGAAAACGTTGCTGGTGTTTTTAATAAGTCTTGTGCTATCTCATACACTGCATACAGAAACGTTTTCCCAATCTGGACATTGGGTAGATTTTCTAGATTGCATCCAAACTCACCATTGGCTGAACATTTGCAATCTAGACCATTGGCAGGTGTTGGTAAAACTCCAAAAACAGCTTTATCAGATTAA |
| TfLss1 | TCAGAACAAAGCCTCATTGCCATACTTTTGAGAAAACAAACCCAATGCTCGGATGGGCCAGTAGAACTTGTAGTTGGGATACGAGATCATACAAGACATGTTGAAAACACCTTCGATCGACTCTTGGAGCCACTCGCCATTTGCCTGTTGTCGGGACATGAGAAGTTTGATTCCTCGGCGGATAGGCTCTGGGTCAGGGTAATCAGCTTCCATAAGAGATAGACACACCCAAGCTGTCTGGACAACTTGGGACATCTCGTGCTGGGTGTAAACGTGCAATTCGCTGCTGAGATACGACTCTCCCCAACCGCCATCTTCCTTCTGCTTGCTCAATAAGAACTCGCATCCACGGCGAGAAGAGTCGCTCGTGCTATAAGTTTCACCAACACTGGAAAGACTTTCAAGCGCAAACAGGGCCGCATAAGTGTAGCAGATACCCCAGTTACCATACCAGCTCCCATCTGGCTTCTGGACGCGTTTGATATATTCGACTGCCTTGTGCTTTGCAGCTTTGATCTCTTCGGCACGATAGTCTGGGTAGAATCTGCTGAAAAGTGACATCGCGGTAATAGATGCAGTCGTACATTCAACGTGATCGTAACTAATCATGATTCCGCCAAACACCTCGGCTGCGTTGAGCCATTCCAGAAGAGGGGAGCCTCGTCTGCTTTCATACTCACTAAAACCACCAGTACTGTTTTGCAGCAAAAGAATACAATCGACCGCATCTTTCAAGCGATCTGCGGAGACAAGTTTAGGAAATCCGTGAATCTCCTGAAGTTGCAAAACCGATCGTAAACCCTCTGCAGTGCAGTCGCTAACCGTATAACCCTGAACCTTATTACTGAAGGGCCAAGCTCCTTTTCTATGTTGACGATAACACTTGTCTTGGTTGGGAACATTTTCACGAAGCTGATGATTATCCAGGAATTCAAGGGCTTTGGTCAGCATTGGTCGCCATTTGGGATCTTCCGCGAAGCCAGCCACAGAAACAGCCTGCGTGATAAAGGCGGTGTCCCAGACTTGAGCGCCGTTGGTGCCATTGCAAAGCATGCCCTCCCCTTTCATCCATAGATATTCGTGTAGCGCCTCTCGGTGTTTTCTCACCGACTCACTTCCTTCACCGTCGTGAATGTAGCAACACACAAAGTTCAGAGGGTTACTGACGGGAGCAAGTCCTGCATAGTCCGTGTTCTCATCCTCCATTCGAATCAATTCCCACACCCAATCTTCAGCTTTCTTGGCCAGCGCCGAAAACCGAAGCAAGGGCGTCCACACGTTCACGAGGAGAGTGTTGACCACGTTGAGAAACATTGTCTTTGGATAGTAATTGTCTTCTTTAGCAATAGAGTTGCGGTGGGAAGCAAAGTCTATACTTTCATAAGGTTGCGCGTATATCTCGTTTCTGAGCTGCCTCGTCAATTCGTTCTGGGGGAAGACCCATTTCTTGGACCACAGGTAAGACATTGGTAAAAACACCATGCGCATGTGAATCCACCATCTATAAGGAGCGATCGGTACCCAGTCAGGAAGTAGCCAAAGCTCCGGTGGCACTGGATTGGCGCAGCTCCAATCCATGACACCCAGAAGAGAAAGCCAGACCTTCGCCCAATGTGGCGCGTAAAGTGCGCCTCCGAGCTTATGAGCAAATCCTCGTGCTTTGATCATTCGAGGATCTTCTTCACTTGCTCCTAGTAAACGCAGGATCACATAGGTCATTACTGTTCCAAATGCAGAGCTATGACCTTCAATATGAAGTCCCCAGCCGCCGTCGACCGGATTTTGTCGCGCAAAGAGATATCGCTTGATTTCCACCGCATACTCGGGAGGTATGGGCGTGTTGGTGATATACCAAGTGATGACTACACCTGGTAAAAGAAACATAGGCCCGCCATACTCGCATGCCCAATGCCCAGTGGGCATCTGTAACTTGGAGAAAAATTCCAGGCCATTGATAGCTGCGTCCAGAGGAGTCTTCGCTTTTGGCAGCTCCGGGGCACCGGTTGGGAGTCCCAGGTTGTATTTGTCGTAAAAGGTTTGAGGCCATTTCTCATTCTCTTCATCGCTTTCCAGATAGCGCCATGTCAAGCGGCCGTCATTATCGTGCAATCTCCACCTTGCATAGTCGGTTTTGTCGTCTCCTGCGGCATCCTTGGTTAGGTGGCCATTGGCATTCGTTCGCCAATTGGCAATGGGCCGGATTTTGCTTTCTTTCGCCAT |
| PbLss1 | ATGACAGTGTATTATTCAGAGAAAATCGGCTTGCCGAAGACTGATCCTCAAAGATGGAGGCTGAGGGTCAATGAGCTCGGACGTCAGTATTGGGATTACATTGAAAAGGAGGACTTGAAGAACGACCCTCAAACACCATACGTCCAGTATCTCTTGAAAGGAGATGAATTTGAATGTCCAATACCTGAAAAACCTCAATCTGCTTTTGAATCGGCCCGAAATTGTGCAGATTTTTTAGCTTTGATTCAAGATGAATCTGGAGTGTTTCCGTGTCAATACAAAGGACCAATGTTCATGAGCATAGGCTACGTTGTAGCATGTTATTTCACTAATACACCAATTCCAGATCATGTAAGGACCGAAATGATCAGATATGTAGTCAATACAGCCCATCCGGTGGATGGAGGCTGGGGACTACACGAGTGGGATAAATCGACATGTTTTGGAACATGTATGAATTACGTTGTCCTTCGATTGTTGGGACTTCCAAAAGATAATCCAGTGTGTATCAAGGCTAGAAAGGTATTACATGCTCTAGGAGGAGCTTTGGCCACTCCATATTGGGGAAAGGCTTGGCTTTCACTTCTAAATGTGTACAAATGGGAAGGAGTGAACCCTGCACCTCCAGAAATGTGGAATCTTCCATACTCGCTCAAAATTCATCCATGTAGATGGTGGGTACATACAAGAGCCATTGCTTTACCACTGAGTTACATTTCTTCCTACAAAAGTCAAATGCCTCTGACTCCACTGTTGAAAGAGCTGAGAAATGAAATATTTCTACAGGATTTCGATACCATAGACTTTTCTAAGCATAGGAACAATGTGTGTGGTATCGATCTTTATTATCCTCATACTTCACTGTTGGACTTTGCGAACAGTATTCTTGTCGGCTACGATAAAATTAGACCTACATGGTTGCTGAAAGAATCTAATGATGCAGTGTATGAGCTGATTAAGAAAGAGCTTGCCAACACAGAGCATCTATGTATCGCTCCTGTGAATGCTGCTTTTAATACGATTGTTGCATACCTTGAAGAAGGTCCAGAGTCGTACAATTTCAAACGCTTGCAAGAAAGATTCAAAGATGTCATCTTCCATGGGCCCCAAGGAATGACCACTATGGGTACCAACGGCACGCAGGTCTGGGACACCAGTTTTTGCCTACAATACTTTTTCATGGCAGGATTAGCAGATCTTGATGAATATGAGGAGTTAATTATCAGAGACAAAAGGATCGGATGCTTTCCATTTAGTACCAAGGAGCAAGGTTACACAGTAAGTGATTGCACTGCCGAGGCAATCAAGGCGATCTTGATGGTGAAAAACCATCCCAAATTTGCCTACCTTGGAGATTACATTGATGAGGATCTGCTTAAGAAAGGTATTGATGGATTATTATCTCTACAAAACGTGGGGTCATATCATTTTGGTTCGTTCTCGACATATGAAAGCACTAGAGCTAATCCTGCGCTGGAAAAGATCAACCCCGCAGAAGTATTTGGCAATATCATGGTAGAGTATCCTTACGTAGAATGTACAGACTCTTCGGTTTTGGGTCTTACCTATTTCAGAGAACATTGGGACTATCGCCGACACGATATTGACACTGCTATAGAGAGGGGTGTCAAGTTCATCTGCGACGCCCAGCAGGAAGATGGGTCATGGTATGGCTGTTGGGGGGTCTGCTTTACTTACGCAGGAATGTTTGCGCTAGAAGCTTTGGCTTCTGTGAATCAATATTATGAAACCAATGAAGTAGTGAGGAAAGGGTGTGATTTCCTGGTCTCCAAGCAGATGGCTGATGGTGGCTGGTCTGAAAGTATCAAATCCTGTGAAACTCATACCTATGTTCGTGGAAAAAGGGGTATGGTAGTTCAAACTTCTTGGGTTCTAATAGGTTTGATTCTTGCTAAGTACCCCCATAAGGAAGTCATAGACAGGGGGGTTCAACTAATCATGAGTCGCCAAAAGACAAGAGGAGACTTTGACTTTGAAGCTGTAGAAGGTATTTTCAACCATAGCTGCGGAATTGAGTATCCAAACTACAAATTCCTGTTTCCCATCAAGGCCTTGGGGTTATATTCCAAAGAGTATGAGCAATAA |

# References

1. Zhu ZT, Du MM, Gao B, Tao XY, Zhao M, Ren YH, Wang FQ, Wei DZ. Metabolic compartmentalization in yeast mitochondria: Burden and solution for squalene overproduction. Metab. Eng*.* 2021;68:232-245.
